# Acute resistance exercise and training reduce desmin phosphorylation at serine 31 in human skeletal muscle, making the protein less prone to cleavage

**DOI:** 10.1038/s41598-024-79385-0

**Published:** 2024-11-14

**Authors:** Daniel Jacko, Kirill Schaaf, Thorben Aussieker, Lukas Masur, Jonas Zacher, Käthe Bersiner, Wilhelm Bloch, Sebastian Gehlert

**Affiliations:** 1 Department of Molecular and Cellular Sports Medicine, Institute of Cardiovascular Research and Sports Medicine, German Sport University, Cologne, Germany; 2 Department of Human Biology, Institute of Nutrition and Translational Research in Metabolism, Maastricht University Medical Centre, Maastricht, The Netherlands; 3 Department of Preventative and Rehabilitative Sports and Performance Medicine, Institute of Cardiovascular Research and Sports Medicine, German Sport University, Cologne, Germany; 4 Department for Biosciences of Sports, Institute of Sport Science, University of Hildesheim, Hildesheim, Germany

**Keywords:** Exercise, Human skeletal muscle, Desmin, Intermediate filaments, Proteins, Cytoskeletal proteins, Phosphoproteins, Skeletal muscle

## Abstract

Desmin intermediate filaments play a crucial role in stress transmission and mechano-protection. The loss of its integrity triggers myofibril breakdown and muscle atrophy for which desmin phosphorylation (_p_Des) is a priming factor. We investigated whether eccentric accentuated resistance exercise (RE) influences the regulation of _p_Des, effecting its susceptibility to cleavage. Ten healthy persons performed 14 RE-sessions (2 per week). Muscle biopsies were collected in both untrained and trained conditions at rest (pre 1, pre 14) and one hour after RE (post 1, post 14). Western blotting and immunohistochemistry were utilized to assess desmin content, phosphorylation at several sites and susceptibility to cleavage. In untrained condition (pre 1, post 1), RE induced dephosphorylation of serin 31 and 60. Trained muscle exhibited more pronounced dephosphorylation at Serin 31 post-RE. Dephosphorylation was accompanied by reduced susceptibility of desmin to cleavage. Additionally, training increased total desmin content, upregulated baseline serine 31 phosphorylation and attenuated pDes at serine 60 and threonine 17. Our findings suggest that acute and repeated RE changes the phosphorylation pattern of desmin and its susceptibility to cleavage, highlighting _p_Des as an adaptive mechanism in skeletal muscle, contributing to the proteostatic regulation in response to recurring stress.

**Supplementary Information:**

The online version contains supplementary material available at 10.1038/s41598-024-79385-0.

## Introduction

Mechanically highly demanded cells or fibers, such as those found in skeletal muscle, rely on an effective network of strain transducing proteins. The muscle-specific type III intermediate filament (IF) protein Desmin plays an important role in this context^1,2^. It is crucial for fiber structure, mechanical integrity, and lateral force transmission^2–4^. Desmin mechanically integrates cellular components by scaffolding Z-discs, thus connecting sarcomeres to each other, as well as to the subsarcolemmal cytoskeleton and organelles such as mitochondria and nuclei to form the complete contractile apparatus^4,5^. In its monomeric state, the protein has a molecular weight of approximately 53 kDa and possesses an IF-typical tripartite organizational structure. This structure consists of a central α-helical rod domain flanked by non-α-helical N-terminal head and C-terminal tail domains^6^. The head and rod domains are important for desmin polymerization to achieve mature IF lengths^7^. In this process, monomers form parallel dimers which then assemble into antiparallel tetramers. Subsequently, eight tetramers construct one unit-length filament. Ultimately, their sequential binding facilitates the longitudinal growth of the intermediate filaments (IFs). The assembly, disassembly, or stability of desmin IFs heavily relies on posttranslational modifications (PTMs), particularly phosphorylation^8–10^, predominantly occurring at sites within the protein’s head domain^11,12^. Although various phosphorylation sites have been identified^8,10,13^, collectively predisposing desmin to disassembly with increased phosphorylation, recent research has highlighted the significance of phosphorylation at serine 31 (_p_Des^S31^)^7,12,14–16^.

For instance, in murine muscle fibers, _p_Des^S31^ has been demonstrated as necessary for IF disassembly, facilitating mitochondria trafficking^15^. Additionally, Makihara et al.^12^ have emphasized the essential role of _p_Des^S31^ in efficient IF separation during mitosis in murine myoblasts. They further illustrated that in vivo, _p_Des^S31^ primarily occurs in muscle cells during early mitosis. Conversely, others^14,16,17^ provided evidence for _p_Des^S31^ presence in adult muscle tissue during pathological conditions. Moreover, recent seminal studies have elucidated the association between desmin phosphorylation and muscle atrophy. Specifically, initial phosphorylation of desmin’s head domain by the glycogensynthase-kinase 3 beta (GSK3-β) is shown to be required for Trim32-dependent ubiquitination^7,18–20^. Subsequent binding of a complex comprising the AAA-ATPase ATAD1 and its interacting partners UBXN4 and PLAA facilitates desmin dissociation from the highly organized myofibrillar complex. This exposes cleavage sites on desmin for calpain 1, ultimately leading to its degradation by the proteasome^21^.

The loss of muscle mass is associated with physical frailty, disability, cognitive decline, and metabolic dysregulation^22–24^. Therefore, its maintenance is crucial for overall health and autonomy of life. Systematic, recurrent resistance exercise or resistance training (RT) is well-studied and widely accepted to increase or maintain muscle mass and to decelerate its loss^25,26^, not only in healthy individuals but also in those with pathologies associated with increased atrophy^27^ like cardiovascular disease^28^ or age-related sarcopenia^29^.

Remarkably, RT also influences desmin content in skeletal muscle. We^30^ and others^31–35^ have shown that high load RT or accentuated eccentric exercise^32^ promptly upregulates desmin content within a few days. However, whether RT affects desmin phosphorylation at which site and which dynamic in untrained and trained healthy skeletal muscle still remains elusive. Previous studies primarily utilize in vitro models or examine muscles in pathological contexts^8,9,14,18,36^. Understanding whether and which phosphorylation site can be specifically modulated by resistance training in human skeletal muscle may enhance our comprehension of desmin-related proteostasis mechanisms, muscle atrophy and non-pharmacological treatment strategies.

Therefore, we aimed to address the following main questions: (1) What is the phosphorylation status of selected phosphosites on desmin IFs in healthy human skeletal muscle under resting conditions? (2) Can acute resistance exercise modify desmin phosphorylation? (3) Does modulation of desmin phosphorylation impact the protein’s susceptibility to cleavage? (4) Does RT influence acute phospho-regulation of desmin or baseline phosphorylation, respectively?

To explore these questions, we administered eccentric accentuated acute resistance exercise and a 14-session training regimen (twice per week) to healthy young men and women. Muscle biopsies were obtained in both the untrained and trained conditions at rest (pre 1, pre 14) and one hour after resistance exercise (post 1, post 14). Desmin content and phosphorylation at serine 31 and 60 (_p_Des^S31^, _p_Des^S60^), as well as threonine 17 and 76/77 (_p_Des^T17^, _p_Des^T76/77^), were analyzed using western blotting and immunohistochemistry.

## Results

### In resting healthy human skeletal muscle, desmin is phosphorylated at several sites

Desmin phosphorylation is a prerequisite for IF disassembly and degradation^18,20,21^. Before, it was reported that _p_Des is a hallmark of especially diseased muscle^14,16,36^. However, a certain turnover of IFs or desmin likely occurs in healthy skeletal muscle as well^7^. This implies that desmin phosphorylation should also be detectable under physiological conditions, although to our knowledge, this has previously not been directly studied, especially in human skeletal muscle.

**Fig. 1:**
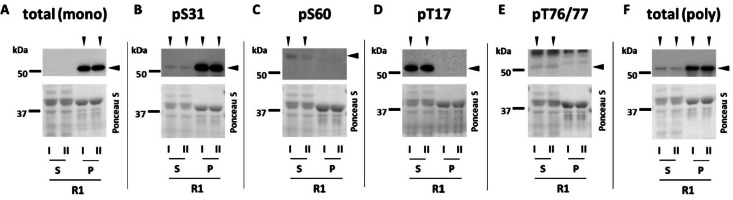
Desmin phosphorylation in untrained condition at resting state (R1) in healthy young human skeletal muscle. Qualitative assessment. Pooled muscle samples (*n* = 3) were fractionated in a detergent (Triton X-100) -soluble supernatant (S) and insoluble pellet (P). Samples were applied as duplicates (I, II) and membranes were incubated with antibodies raised against desmin phosphorylated at serine 31 (pS31) (**B**), 60 (pS60) (**C**), threonine 17 (pT17) (**D**), 76/77 (pT76/77) (**E**) as well as total desmin (**A**, **F**; two different antibodies; one monoclonal [mono] and one polyclonal [poly]). Ponceau S was used as loading standard. Vertical arrowheads indicate bands or fraction positive for respective antibody. Horizontal arrowheads indicate the analyzed bands. It is to note that the _p_Des^S60^ antibody recognized a single band which however appeared slightly above the other bands (see text). For _p_Des^S76/77^, only bands at predicted height were considered.

Therefore, as a first step, we investigated whether desmin phosphorylation could be detected in skeletal muscle from healthy young adults at resting state (pre 1). Additionally, given that desmin is found in both the insoluble cytoskeletal fraction and the soluble cytoplasmic fraction^17,36^, we tested total desmin (Fig. 1A, F) and phospho-specific antibodies (Fig. 1B-E) on both compartments (supernatant (S) and insoluble pellet (P)).

Our results revealed that desmin is phosphorylated at rest at all four investigated sites (S31, S60, T17, and T76/77) (Fig. 1). Interestingly, the observed phosphorylation sites showed a specific subcellular localization. While _p_Des^S31^ (Fig. 1B) and _p_Des^T76/77^ (Fig. 1E) were detected in both the pellet and the supernatant (with the majority in the pellet for S31), the two other sites, _p_Des^S60^ (Fig. 1C) and _p_Des^T17^ (Fig. 1D), were exclusive to the supernatant. This finding was somewhat surprising, as the monoclonal anti-total desmin antibody, raised against carboxy-terminal residues, only detected desmin in the pellet (Fig. 1A). To confirm the specific localization of desmin in the supernatant, we tested a polyclonal antibody recognizing carboxy-terminal residues, which indeed confirmed its presence in this fraction (Fig. 1F). We therefore speculate that the inability of the monoclonal antibody to recognize the supernatant-localized desmin pool may be due to epitope masking. The supernatant or cytosolic desmin pool, being detergent-soluble, might undergo post-translational modifications that hinder antibody binding. Desmin is known to undergo various PTMs^10^, such as ubiquitination^20^ or, as recently shown O-GlcNAcylation^37^. Interestingly, the latter occurs exclusively in the nucleus and cytosol^38^, which could explain another phenomenon we observed: the antibody against _p_Des^S60^ produced a band slightly higher than predicted or than the total protein (Fig. 1C). As it was a single band on the whole Western blot membrane and no additional bands occurred, we believe it to be a specific signal. To explain the higher molecular weight pattern, we follow the above-mentioned modification argument. It is well known that these PTMs can alter band appearance in SDS-PAGE or Western blotting by adding mass or altering electrophoretic mobility^39^. In this case, O-GlcNAcylation might be a candidate, as this type of modification is restricted to the nucleus and cytosol^38^, and the pS60 antibody detection was exclusively in the cytosol. Although plausible, it remains a limitation as we cannot prove it experimentally.

In summary, our findings suggest that desmin phosphorylation is not exclusive to diseased muscle but also occurs as a regular physiological mechanism in healthy muscle. Considering the different subcellular localizations, and to encompass the entirety of myofibrillar desmin, we opted to perform subsequent analyses on whole cell lysates.

### Phosphorylation state of desmin can be specifically influenced by acute resistance exercise

**Fig. 2:**
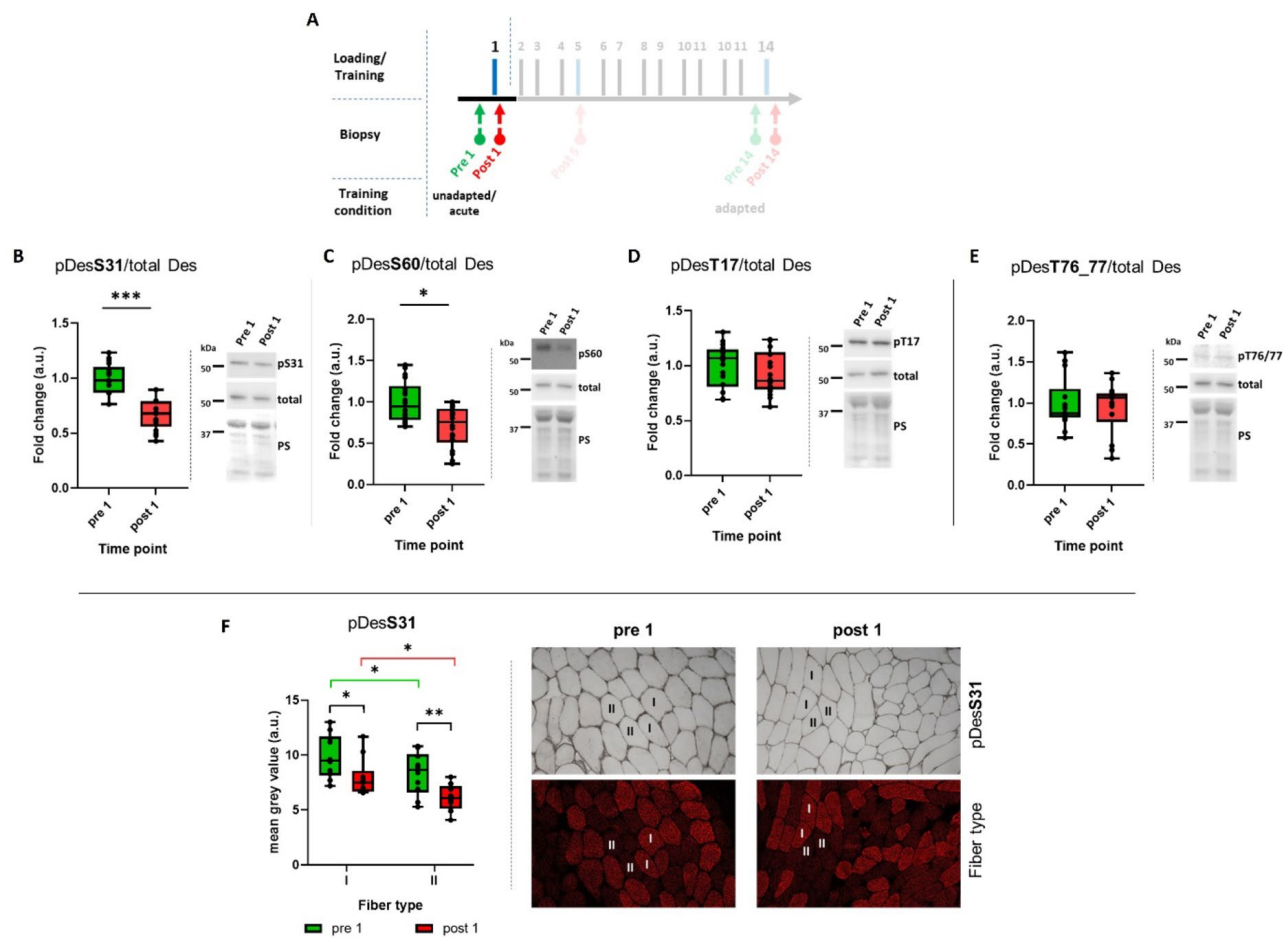
Desmin phosphorylation (pDes) following acute resistance exercise in untrained conditions. (**A**) Extract of the study design highlighting the time points relevant to the data presented in this figure. (**B**–**E**) Box plots of western blot results based on 18 subjects for desmin phosphorylation at serine 31 (S31), 60 (S60) and threonine 17 (T17), 76/77 (T76/77). Right panels beside the graphs show representative images of western blots and membranes stained with Ponceau S (PS), which was used as loading control. (**F**) Box plots of immunohistochemical (IHC) results based on 10 subjects for pDesS31differentiated in type I and type II muscle fibers. Right hand panels show representative images of IHC stainings. *p < 0.05; **p < 0.01; ***p < 0.001; *n* = 18 (**B**–**E**), *n* = 10 (**F**). The cropping and merging of images are indicated by delineation with dividing white space.

To assess whether the phosphorylation status of intermediate filaments (IFs) could be specifically influenced, we applied controlled stress via resistance exercise to the muscles of previously untrained subjects and obtained muscle biopsies before at rest (pre 1) as well as one hour post-loading (post 1)(Fig. 2A). As shown in Fig. 2B-E, acute resistance exercise led to the dephosphorylation of desmin at the two analyzed serine sites, 31 and 60 (*p* < 0.001 and *p* < 0.01), while threonine 17 and 76/77 remained unaffected. To support these findings, we also performed immunohistochemistry (IHC) analysis, which served the additional purpose of providing insight into potential fiber-type-specific regulation patterns (Fig. 2F). In IHC-stained muscle cross sections, we determined that _p_Des^S31^ was dephosphorylated due to acute resistance exercise, consistent with the results of the Western blot analysis. Furthermore, we found that type I fibers generally displayed a higher level of _p_Des^S31^ (*p* < 0.05). However, this finding is consistent with the higher abundance of total desmin in type I compared to type II fibers^40^.

### Resistance exercise-induced dephosphorylation of desmin protects the protein from cleavage

A higher phosphorylation state of desmin makes the protein prone to calpain-dependent cleavage^18,19^. Conversely, a reduction in desmin phosphorylation should attenuate the susceptibility of desmin to cleavage. Given this background, we were next interested in whether RE-induced dephosphorylation would have a corresponding physiological consequence.

**Fig. 3:**
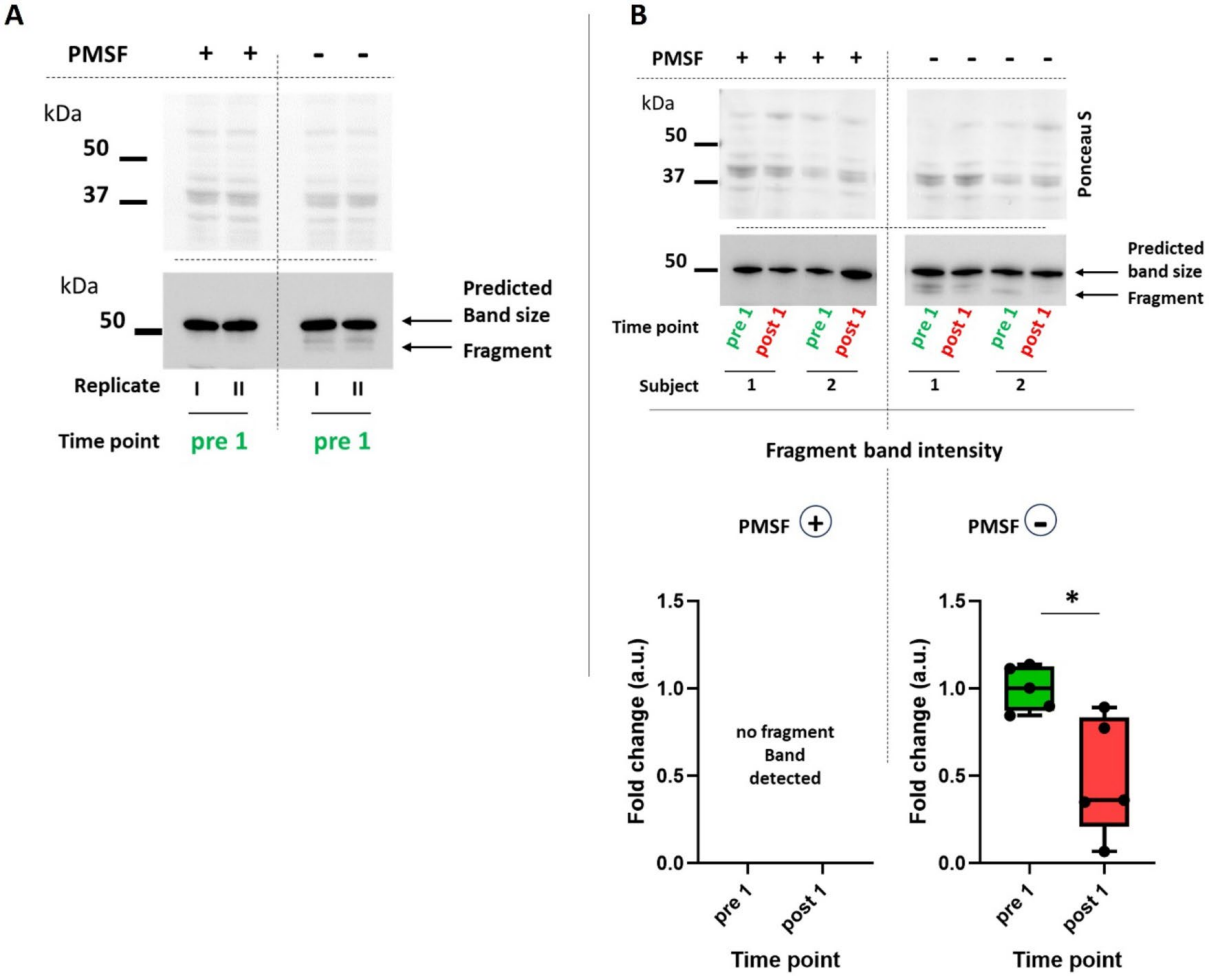
Cleavage band appearance following omitting the protease-inhibitor PMSF during tissue lysis in resting (pre 1) as well as acutely exercised skeletal muscle (post 1). (**A**) Western blot image with pooled muscle samples (*n* = 4) at resting state (pre 1) applied as duplicates (I, II). The left panel shows samples lysed with the addition of the protease inhibitor PMSF (other protease inhibitors were also included, see methods). The right panel shows samples lysed by omitting solely PMSF. Here, the appearance of a fragment band just below 50 kDa was obvious. The top panel shows the same membrane, stained with Ponceau S, what was used as loading control. (**B**) Comparison of fragment band intensity between samples from 5 subjects taken at resting state (pre 1) and one hour after acute resistance exercise (post 1). Lower panel: Box plot showing quantification of fragment band intensities. (left side) Control samples (PMSF +): No fragment band appearance, thus no quantification was possible. (right side) Samples without PMSF (PMSF–). *p < 0.05. The cropping and merging of images are indicated by delineation with dividing white space.

For this purpose, we employed a straightforward approach. We performed muscle tissue lysis in duplicate: one set was lysed under standard conditions with all protease inhibitors included, while the other set was lysed by omitting the potent protease inhibitor PMSF (while other protease inhibitors remained included, see methods). To assess whether the absence of PMSF would induce desmin cleavage, we initially used pooled resting state muscle samples from untrained subjects (pre 1). As depicted in Fig. 3A, omitting PMSF resulted in an additional band of approximately 49 kDa, which is below the main predicted size (53 kDa). This band type has been identified in previous publications as a calpain 1-dependent cleavage product of desmin^16,18,36,41–43^. It is worth noting that with prolonged exposure time, this fragment band also appears in some subjects, even with complete protease inhibitor or PMSF addition. This has also been reported by others^16,36,43^. However, we found that omitted PMSF significantly increases this effect.

Subsequently, we applied the same methodology to muscle samples obtained both, before (pre 1) and after (post 1) exercise in untrained condition, and assessed the intensities of fragment bands. As illustrated in Fig. 3B, in acutely exercised muscle (post 1), where desmin exhibited reduced phosphorylation, the protein demonstrated also significantly less fragmentation compared to the resting state (pre 1), when more phosphorylated. This suggests that the observed exercise-induced decrease in phosphorylation possibly renders desmin less susceptible to cleavage.

### Resistance training status affects the acute resistance exercise-induced phosphorylation state of desmin

Skeletal muscle is a highly adaptable tissue, and besides muscle growth, resistance training can increase sarcomeric cytoskeletal proteins like filamin C^44^ or importantly, total desmin content^30,32,33,35,45^. Furthermore, we and others have shown that training alters exercise-induced phosphorylation of various proteins such as ribosomal protein S6, p70S6K1, α B-crystallin, etc^30,46,47^. Therefore, we were interested in whether the physical training condition would also affect post-exercise _p_Des.

**Fig. 4:**
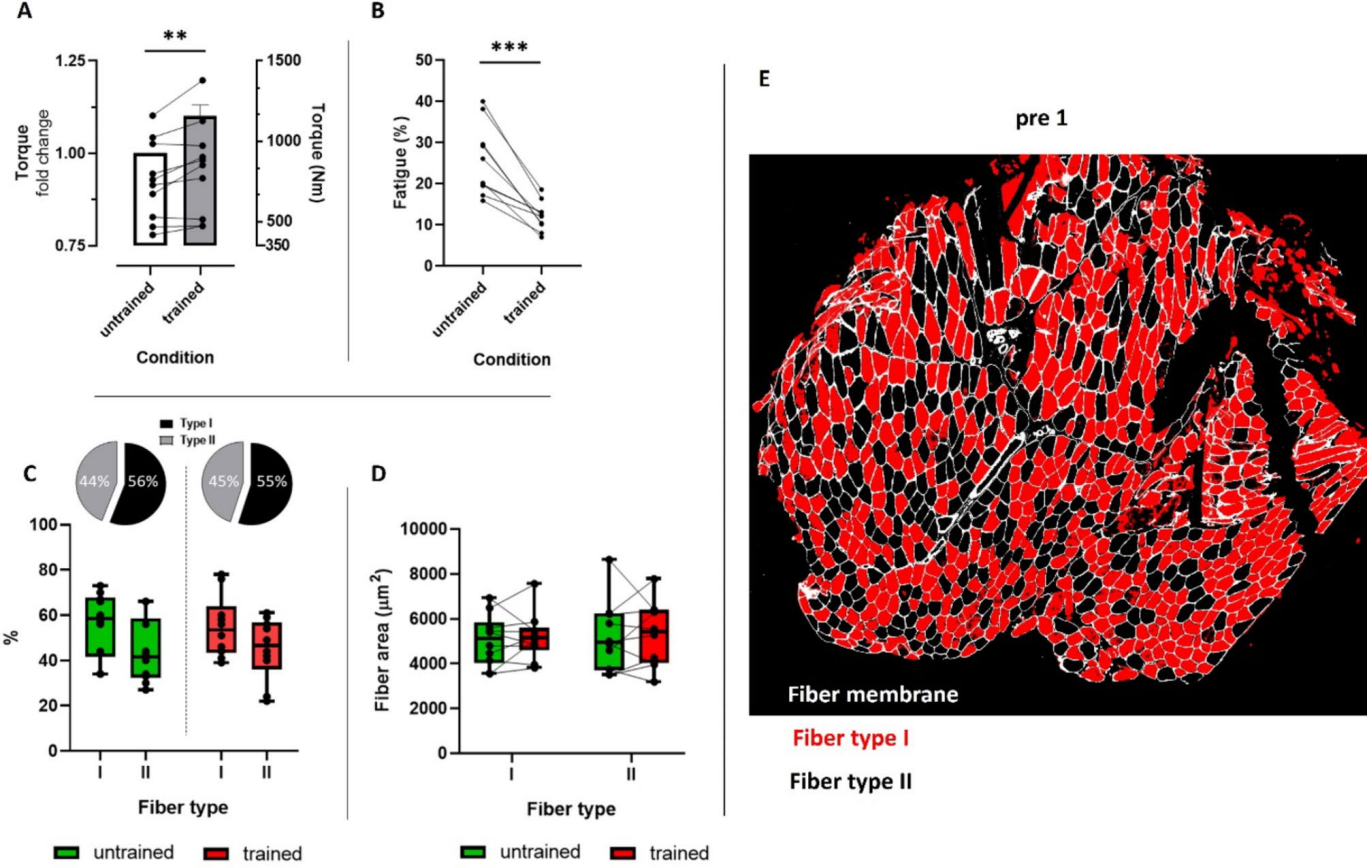
Training induced functional (**A**, **B**) and morphometrical (**C**, **D**) adaptations in skeletal muscle. (**A**) Mean fold change of the maximal isometrically generated torque of the knee extensors (left y-axis; bar graph) in combination with individual absolute torque values (right y-axis; line graph), (**B**) relative exercise induced muscle fatigue, (**C**) type I and type II muscle fiber distribution as well as (**D**) cross-sectional area in untrained and trained condition. (**E**) Representative image of immunohistochemical staining used for determination of type I and II muscle fiber distribution and size before (pre 1, untrained) and after training (post 14, trained). As no differences were found between training states, only pre 1 is shown. The statistics in (**A**) refer to the fold change data. Error bar represents SEM. **p < 0.01; ***p < 0.001; *n* = 10.

In a first step, we ensured that the training applied was effective by demonstrating adaptations in muscle force (*p* < 0.01); (Fig. 4A) and fatigue resistance (*p* < 0.001; Fig. 4B) without altering fiber type distribution (Fig. 4C, E)or radial growth (Fig. 4D, E).

**Fig. 5:**
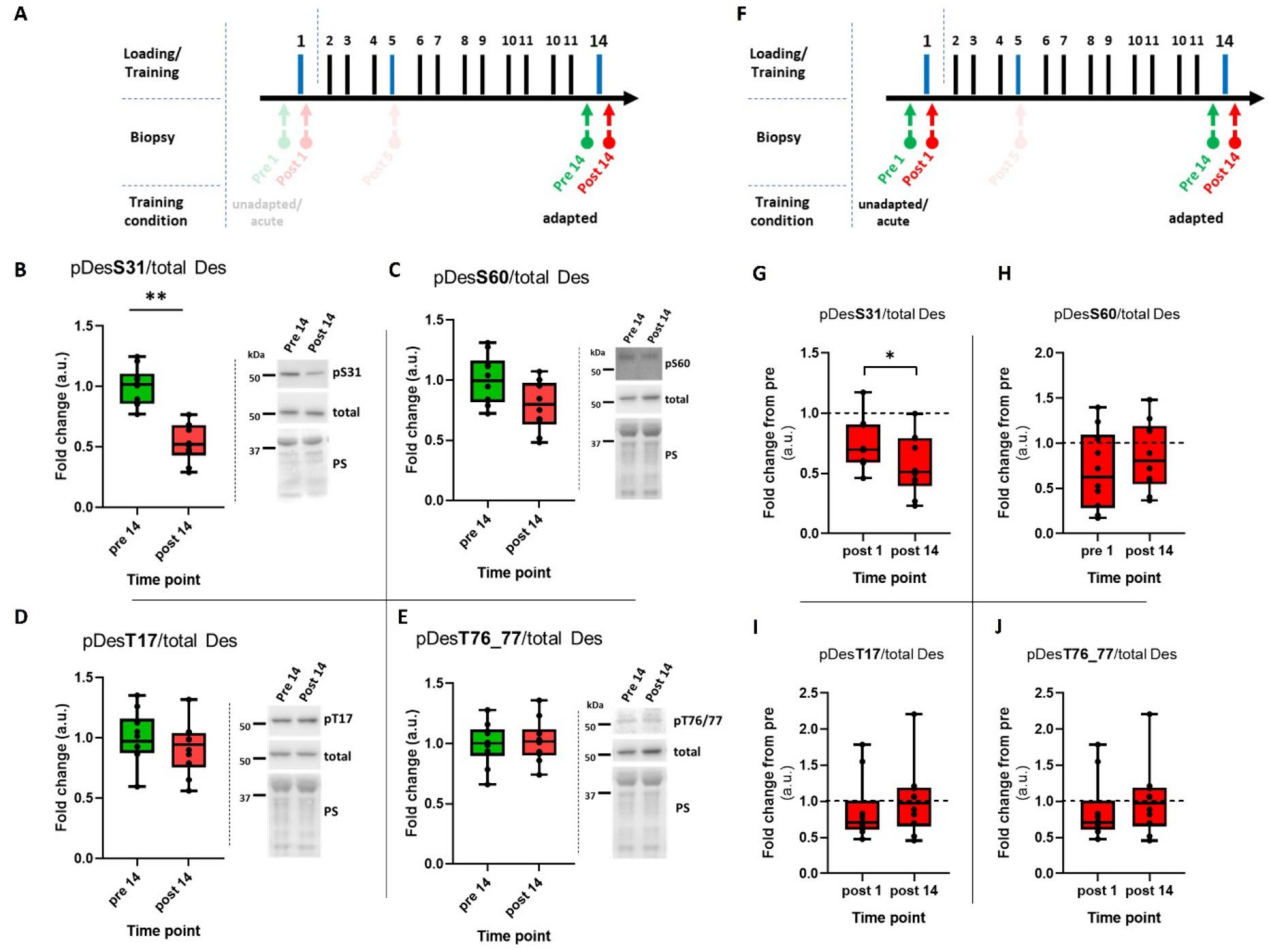
Effect of resistance training on phosphorylation status of desmin (pDes). (**A**–**E**) pDes following resistance exercise in trained condition (pre 14, post 14). (**A**,** F**) Extracts of the study design highlighting the time points relevant to the data presented in this figure. (**B**–**E**) Box plots of western blot results for desmin phosphorylation at serine 31 (S31), 60 (S60) and threonine 17 (T17), 76/77 (T76/77). Right panels beside the graphs show representative images of western blots and membranes stained with Ponceau S (PS). (**G**–**J**) Comparison of acute exercise-induced regulation of pDes between untrained (post 1) and trained condition (post 14). Post 1 was normalized to pre 1 and post 14 was normalized to pre 14. *p < 0.05; ***p < 0.001; *n* = 10. The cropping and merging of images are indicated by delineation with dividing white space.

Next, we investigated whether the exercise-induced regulation of pDes would be altered in trained conditions compared to untrained conditions (Fig. 5A, F). In the trained condition, we observed a significant downregulation of phosphorylation at S31 as a result of acute resistance exercise (*p* < 0.01; Fig. 5B). Furthermore, this downregulation was more pronounced in the trained than in the untrained condition (*p* < 0.05; Fig. 5G). The remaining three sites showed no effect of acute resistance exercise and did not differ in their regulation between physical training states (Fig. 5C-E and H-J).

### Resistance training affects total desmin content and its baseline phosphorylation status

**Fig. 6:**
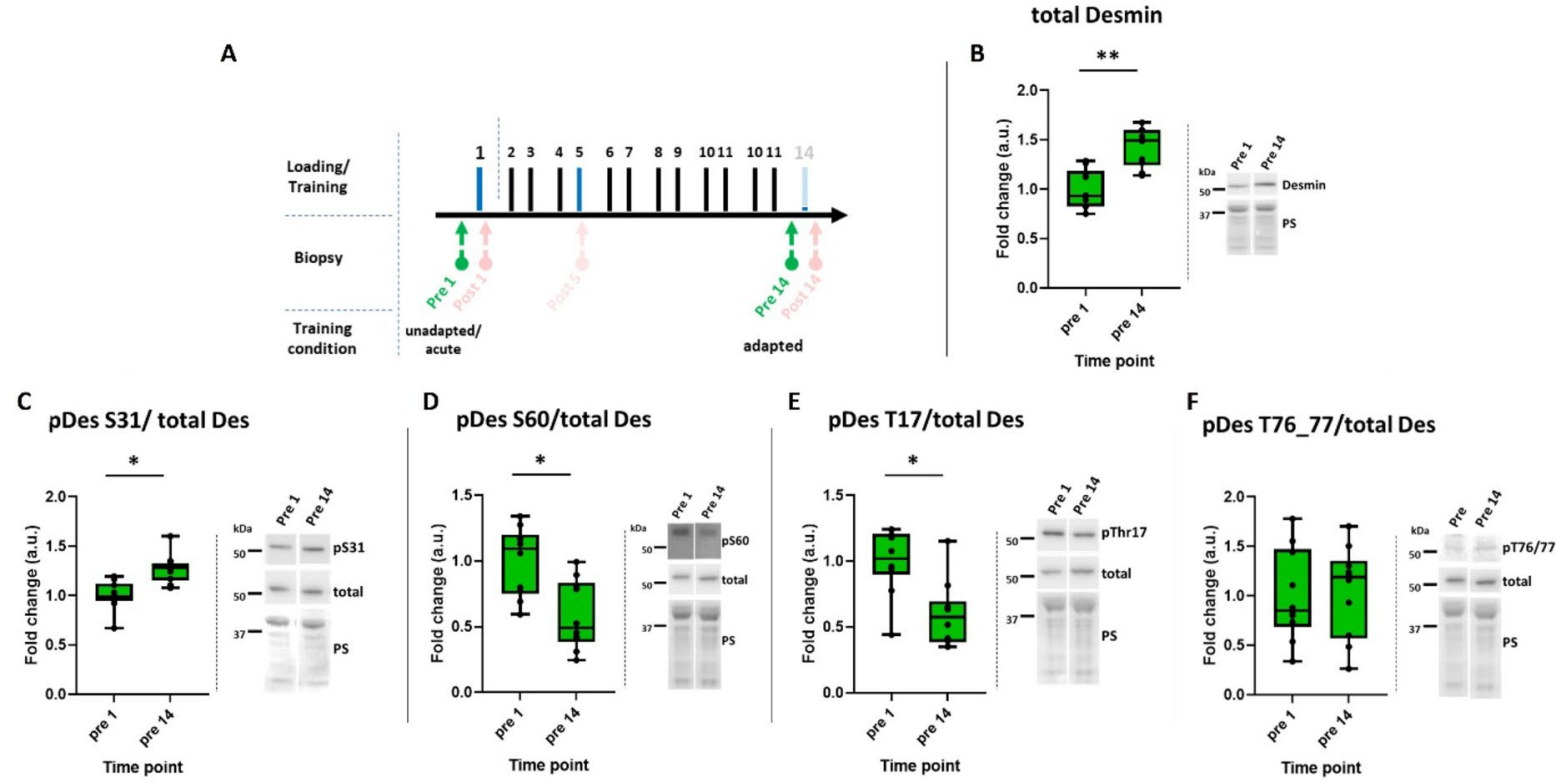
Baseline desmin phosphorylation following acute resistance exercise in trained condition. (**A**) Extract of the study design highlighting the time points relevant to the data presented in this figure (pre 1, pre 14). (**B**) Box plots of western blot results for total desmin. (**C**–**F**) Box plots of western blot results for desmin phosphorylation at serine (S) 31, 60 and threonine (T) 17, 76/77. Right panels beside the graphs show representative images of western blots and membranes stained with Ponceau S (PS). *p < 0.05; *n* = 10. The cropping and merging of images are indicated by delineation with dividing white space.

In previous publications, we^30^ and others^31–35^ have shown that resistance training results in an increase in total desmin content. In the present study, we were able to confirm this as proof of principle; total desmin content increased from pre 1 to pre 14 as a consequence of training (Fig. 6A, B). Training also altered the baseline (pre) phosphorylation status of the protein (pDes normalized to total Des). Specifically, pDes at S31 was significantly increased (Fig. 6C), while S60 (Fig. 6D) and T17 (Fig. 6E) were decreased at the resting state due to training (*p* < 0.05). There was no change for pDes T76/77 (Fig. 6F).

## Discussion

We have investigated whether acute RE and continued resistance training will impact desmin phosphorylation and as a functional consequence also affect desmin cleavage. We showed that: (1) Desmin phosphorylation at several sites is a constitutive state in healthy muscle. (2) This phosphorylation state can transiently be specifically influenced by acute RE, resulting in a dephosphorylation of desmin. (3) The acute exercise, and most likely the dephosphorylation of desmin caused by it, renders the protein less prone to cleavage. (4) Chronic contractile stress induced by RT increases total desmin content, augments the acute exercise-induced dephosphorylation and alters the baseline phosphorylation state of the protein differentially, i.e.: tunes up _p_Des^S31^, while downregulating _p_Des^S17^ and _p_Des^T60^.

Before, desmin phosphorylation has primarily been studied in vitro^8,9,12^ and in a pathophysiological contexts such as ischemic heart failure^12,14,16,17^ denervation, and catabolic states^20,43^. In these conditions, increased desmin phosphorylation is linked to IF-System destabilization, depolymerization, and amyloid-like oligomer formation^14,16,18,48^. Thus, especially _p_Des^S31^ and other sites like S60, T17, and T76/77, were regarded as hallmarks of pathophysiology or diseased muscle^10,14,16^, particularly in cardiac^14,36^ and animal skeletal muscle^43^.

Here we show that desmin phosphorylation occurs regularly in healthy human skeletal muscle, a dynamic tissue constantly remodeling, even in the absence of stress. According to Agnetti et al.^7^, the desmin-IF system is subject to this ongoing remodeling process through constant depolymerization and reconstruction by new monomers, too, maintaining IF integrity and cellular homeostasis. Furthermore, mitochondrial trafficking, an ongoing process in muscle, depends on desmin-IF disassembly, driven by _p_Des^S31^^15^. Hence, the constitutive PTM of desmin might facilitate this dynamic exchange.

However, it cannot be excluded that the degree of phosphorylation in diseased or aged skeletal muscle is notably higher than what we have seen here in healthy muscle. Indeed, some studies performed on failing hearts of dogs and humans^14^ and in mouse skeletal muscle due to denervation and fasting^43^, suggest this. Yet, especially for human skeletal muscle, there is a lack of data, and this needs to be elucidated in upcoming research. Nevertheless, our observations provide a new perspective on the PTM of IFs, considering its role under physiological conditions.

Despite evidence linking desmin phosphorylation to muscle cell or fiber homeostasis, no in vivo strategy exists, to modulate it, as noted by Agnetti et al.^7^. However, influencing desmin phosphorylation or dephosphorylation could be a promising strategy to counteract muscle wasting. We demonstrate that acute RE transiently decreases desmin phosphorylation or dephosphorylation. To our knowledge, we are the first to show a possibility of targeted intervening in the regulation of specific phospho-sites of desmin-IFs.

Phosphorylation of desmin preferentially occurs in its head domain, and an overall increase in phosphorylated desmin is associated with loosening of the IFs structure, making desmin accessible for ubiquitination, calpain-dependent cleavage, and degradation^7,18,21^. In the present study, the two serine sites 31 and 60 were sensitive to acute RE. Previously, the sites were associated with myoblast fusion and cell division^10,13^, IF-disassembly^15^ and amyloid-like oligomer formation^14,16^, further highlighting their influence on IF stability by modulating desmin reorganization^12,49^.

While systematic RT unequivocally has positive effects on muscle function, mass, and overall health^25–27^, due to the high mechanical strain, acute RE can also induce myofibrillar damage, especially in an unadapted state and when eccentric contractions are conducted^50–52^. Such myofibrillar damage is characterized by Z-line streaming, membrane damage, and disrupted cytoskeletal organization^53–55^. As desmin-IFs play a crucial role in orchestrating the cytoskeletal integrity as a main strain-transmitting component, it is not surprising that the protein is affected by acute, unaccustomed RE. In this context, a loss of desmin immunostaining has been noted following eccentric loading of the tibialis anterior muscle in rats^31,56^ and rabbits^52^, indicating degradation of the protein. Thus, our finding of reduced desmin phosphorylation one hour post-acute RE was surprising, as an increase in phosphorylated desmin is typically expected for protein degradation, based on the aforementioned studies. The opposing findings may imply the following: a reduction in phosphorylation as a result of acute loading might transiently protect the IF system from destabilization during phases of increased stress. This is supported by the aforementioned studies, showing an initial loss of desmin immunostaining shortly after exercise^52^, which intensified and peaked after 12 h^31^. Nevertheless, to better understand this, future studies should examine desmin’s PTM over a 24-hour period.

Furthermore, it raises the question of the molecular mechanism for the reduction in phosphorylated desmin signal following acute RE: is it a result of decreased phosphorylation or increased dephosphorylation? Recently, Aweida et al.^18^ demonstrated that desmin is a substrate of glycogen synthase kinase 3-β (GSK3-β). GSK3-β-dependent phosphorylation of desmin was shown to prime the protein for depolymerization and subsequent myofibril disassembly or atrophy. Conversely, inhibition of GSK3-β prevented desmin phosphorylation, depolymerization, and atrophy^18,21^. Although we did not assess GSK3-β activity or phosphorylation in the present study, our findings of reduced phosphorylation following acute RE can be explained by previous studies covering GSK3-β regulation as a result of exercise. These studies show a decrease in GSK3-β activity following submaximal and maximal endurance-type exercise, downhill running, and passive stretch in rodent^57–60^ as well as human skeletal muscle^61^. GSK3-β is constitutively active and can be deactivated upon phosphorylation at serine 9 by protein kinase B (PKB/Akt)^62,63^. PKB/Akt activity, which relies on phosphorylation at T308 and S473, is also sensitive to exercise-induced stress^60,61,64,65^. It is conceivable that desmin undergoes perpetual phosphorylation by constitutively active GSK3-β. Subsequently, exercise-induced activation of the PKB/Akt axis may phosphorylate and deactivate GSK3-β. Consequently, this cascade is suggested to influence or decrease desmin phosphorylation, potentially through a more dominant influence of phosphatase activity.

Taken together, the discussed signaling connections establish a plausible link between physical activity, the activation/deactivation of key kinases, and the observed reduction in desmin phosphorylation following acute RE. However, empirical evidence is needed to substantiate these connections in future studies.

Increased desmin phosphorylation is associated with a higher susceptibility of the IFS to calpain 1-dependent cleavage, and a well-established mechanism proposes the following sequence of events: initial phosphorylation of desmin by GSK3-β is necessary for facilitating Trim32-dependent ubiquitination^19^. Subsequently, ATAD1 binds phosphorylated and ubiquitinated desmin IFs, along with its interaction partners PLAA and UBXN4, leading to the dissociation of IFs. This process extracts desmin from the tight IF-network, exposing calpain 1-specific cleavage sites on desmin. Consequently, calpain 1 cleaves desmin, rendering it accessible to the ubiquitin-proteasome system for degradation^21^.

When increased desmin phosphorylation makes the protein more prone to cleavage and degradation, conversely, decreased phosphorylation should reduce this susceptible. Our findings support this: muscle previously subjected to acute RE, with reduced desmin phosphorylation, exhibited lower susceptibility to cleavage compared to resting muscle with higher levels of phosphorylated desmin.

Desmin fragmentation or cleavage has been extensively studied, with Nelson and Traub^42^ providing a detailed description of the characteristic fragment band pattern of purified desmin cleaved by calpain 1. Notably, the N-terminal part is particularly vulnerable and is cleaved rapidly by Ca^2+^-dependent proteases, resulting in a small and a large fragment with rod- and tail-domain comprising approximately 49 kDa^42^. Interestingly, the small 9 kDa N-terminal fragment is crucial for the assembly of 10 nm desmin filaments and for their binding to nucleic acids^42,66^. This indicates that the removal of this part by the protease is not just a matter of desmin turnover but rather serves the regulation of specific cell functions^42^. The fragmentation, particularly the approximately 49 kDa fragment, has been observed in numerous studies^16,18,36,41,43^.

By demonstrating that acute RE reduces desmin phosphorylation, leading to decreased susceptibility of the protein to cleavage of the N-terminal fragment, we establish a link between contractile activity, its influence on desmin modification, and the corresponding functional consequence. However, it is essential to consider several factors. First, we have not confirmed whether cleavage occurs in vivo or is solely a consequence of cell lysis. Second, it remains unclear whether in our study, in fact the head domain is specifically targeted for cleavage. While our assumptions align with previous evidence, we lack experimental confirmation. Nevertheless, the lack of recognition of the 49 kDa band by the phospho-antibodies raised against the N-terminal domain lends support to our hypothesis.

As previously discussed, acute unaccustomed exercise can have detrimental effects on skeletal muscle, while systematic training unequivocally has positive adaptive effects. To validate the effectiveness of our training regimen, we demonstrated an increase in muscle force and fatigue resistance (Fig. 4A and B), without affecting muscle fiber type composition or size (Fig. 4C and D). Concurrently, we observed a significant upregulation in total desmin content (Fig. 6B). This increase following resistance training has been previously reported by our group^30^ and others^32,33,35,45^. Notably, an elevated desmin content may contribute to enhanced resilience of skeletal muscle fibers against mechanical stress^30^. Moreover, the increase in desmin content, despite the absence of fiber hypertrophy, has been reported previously^30^. This dynamic nature of desmin suggests that it surpasses most other proteins involved in fiber hypertrophy. While loss of desmin is considered a prerequisite for muscle atrophy, an increase in desmin content may not necessarily precede muscle hypertrophy. This was demonstrated by Joanne et al.^67^, who showed that mice subjected to one month of RT, regardless of desmin knockout or wild-type status, exhibited similar increases in muscle weight. Thus, muscle hypertrophy can occur independently of desmin content increase, and vice versa, suggesting that these adaptive mechanisms are not interdependent.

In this study, we demonstrated that desmin is phosphorylated at all four investigated sites in resting skeletal muscle (Fig. 1). Interestingly, RT affected baseline phosphorylation, upregulating S31 and downregulating while S60 and T17 in the trained state (pre 14) compared to the untrained state (pre 1; Fig. 6). However, the functional significance of these altered phosphorylation patterns remains unclear, with no current studies offering precise conclusions.

Yet, considering only _p_Des^S31^ (Fig. 5B) and following our previous argumentation, the increased baseline phosphorylation in the trained state might suggest an even higher susceptibility of IFs to destabilization and cleavage compared to the untrained state. This seemingly contradictory observation is hypothesized to indicate a mechanism that enhances the dynamics of the desmin-IFs, thereby promoting adaptation processes in this system. Additionally, we observed a reduction in phosphorylation of S31 following acute exercise in the trained state that was significantly more pronounced as compared to the untrained state. As discussed earlier, this acute exercise-induced dephosphorylation is considered to transiently protect the IF system from destabilization during periods of increased stress. Thus, the augmented acute loading-induced dephosphorylation in the trained state could represent a heightened adaptive response, providing greater protection for IF integrity.

However, this interpretation overlooks the behavior of baseline _p_Des^S60^ and _p_Des^T17^ in the trained state which, unlike S31, displayed reduced phosphorylation. The functional implications of this divergence are challenging to interpret, as previous studies have typically assumed a uniformly directed modification^13^, they did not differentiate into specific sites^17,18,20,21,36,43^ or focused on one site only^12,15,16^. At present, we can only speculate that the differentially altered baseline phosphorylation of desmin may be associated with changes in protein turnover, potentially facilitating protein accrual. However, further research is needed to elucidate the underlying mechanisms and functional consequences of these phosphorylation changes for skeletal muscle.

In summary, our research highlights the importance of regulating _p_Des under physiological conditions in human skeletal muscle, focusing on non-pathological aspects of desmin-PTM. We demonstrated that acute RE transiently decreases desmin phosphorylation, rendering it less susceptible to cleavage. Furthermore, RT alters acute exercise-induced phospho-regulation as well as the protein’s baseline phosphorylation state. However, the functional significance of these alterations requires further elucidation. Furthermore, as we used eccentrically accentuated RE, it remains to be clarified how specific the observed regulations are for this loading mode, or which loading modes are most effective to reduce desmin phosphorylation and render the protein less susceptible to cleavage.

In conclusion, our study underscores the effectiveness of acute RE and prolonged training in modulating desmin phosphorylation and the integrity of the protein within skeletal muscle. This modulation appears to play a crucial role in preserving proteostasis under both, acute and chronic stress conditions. However, further exploration into the underlying mechanisms and fundamental regulation of desmin PTMs under physiological conditions is warranted. Such insights are essential for the development of targeted interventions aimed at combating muscle atrophy processes and enhancing overall skeletal muscle health.

## Methods

The protocols used in the present study were permitted by the ethics committee of the German Sports University Cologne (application #005/2018) and comply with the declaration of Helsinki. We confirm that all methods and experimental protocols were performed in accordance with the relevant guidelines and regulations. All subjects were informed verbally and in written form about the purpose of the study and risks involved before providing written informed consent to participate in this study.

### Subjects

**Fig. 7:**
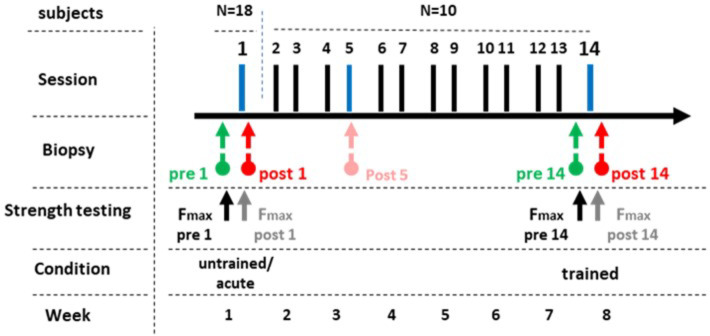
Study design. In a first part of the intervention, 18 at that point resistance untrained participants were subjected to acute resistance exercise. Skeletal muscle biopsies were taken at rest (pre 1) and one hour after completion (post 1). Then, ten of the initial 18 participants continued the training, performing two sessions per week and in total 14 resistance exercise sessions. Finally, now in trained state, biopsies were taken at rest before the 14th session (pre 14), as well as one hour afterwards (post 14). It is to mention, that another biopsy was taken after the 5th session, which however is not included in the present study. Testing of maximal isometric force was conducted after the warm up before the exercise (pre 1, pre 14) as well as immediately after completion of leg extensions and leg press (post 1, post 14).

We recruited healthy, active, but not exceptionally resistance- or endurance-trained participants. Finally, 18 male and 4 female subjects participated (24.7 ± 3.5 yrs; 178.6 ± 9.9 cm; 75.9 ± 12.6 kg) in the study. All 18 subjects participated in the first part of the intervention (Fig. 7). The second part was conducted by a subset of 10 subjects (7 male and 3 female; 25.1 ± 3.4 yrs; 177.3 ± 90 cm; 74.3 ± 15.5 cm).

### Resistance training procedures

All subjects performed an RT of the lower body, with eccentric accentuation (see below). Desmin is a main force transmitting component of the cytoskeleton and previous work indicated, that mechanical tension is a relevant factor to modulate it. Thus, the aim was to emphasize the mechanical component of the contraction-induced stress.

At the start of every RT session, an initial warm-up phase was conducted consisting of two sets of 10 bodyweight squats and one set of 10 repetitions (reps) of leg extensions with 70% of the 8 RM. Afterwards, the main resistance exercises were performed on leg extension and leg press machines in the following pyramid-scheme: 2 sets of 4 reps with the 4-repetition maximum (RM), 2 sets of 8 reps with the 8 RM and one set of 8 reps with 70% of the 8 RM, in which the concentric phase was performed with both legs, the eccentric phase alternately with only a single leg. This aimed to emphasize the eccentric work. Finally, two sets of 16 reps were performed on the leg extension machine only. The resting time between sets and exercises lasted two min and the contraction pattern was 1 s concentric, 1 s isometric, 2 s eccentric and 1 s isometric. After that, two sets of drop-jumps were performed with 10 reps per set from a height of 60 cm. The touchdown was followed by a 1s isometric phase without subsequent takeoff. Finally, three sets of downstair walks over 7 floors, in total 182 stairs, were performed. With each step, 2 stair treads were descended at a time. The subject reached the top floor by elevator.

It is to note that in the following, we will speak of resistance exercise (RE) when it involves a one-time, acute load. Resistance training (RT), on the other hand, means the systematic and regular repetition of the load over the course of several weeks.

### Intervention

The intervention was preceded by a familiarization day where subjects trained with the respective training machines, learned the contraction pattern and the individual training weight for each machine was determined.

The main intervention started the following week, at least 5 days after the testing day by collecting baseline biopsies (pre 1) between 8:00 AM and 10:00 AM. At least 3 days after baseline biopsies, the first RE session with subsequent biopsy (post 1) was performed between 7 AM and 10:30 AM. All post biopsies were taken 60 min after the last contraction of the RT procedure.

In the following 7 weeks, participants performed the RT two times per week. The RE performed within the training intervention followed the same protocol, training load and volume throughout the intervention period. Before and after the 14th last training session pre- and post RE-biopsies (pre 14 and post 14) were collected once again according to the same pattern as described above.

Furthermore, the maximum isometric force (torque in Nm) of the leg extensor at a fixed knee angle of 120° was assessed during both the initial and 14th training sessions post-warm-up, as well as following each exercise. The participants were positioned upright in the leg extension machine, which was equipped with a force sensor (S-Beam, KM1506 K 5 KKN, Megatron) and instructed to apply an initial force of 50 Nm to the lever arm pad. The contraction was intended to be explosive and sustained for 3–4 s. Participants were permitted to grasp the seats grips to prevent their hips form lifting off the seat.

### Biopsies

Biopsy collection took place in the Outpatient Clinic for Sports Traumatology of the German Sports University Cologne (Cologne, Germany). In total, four biopsies were taken from the *musculus vastus lateralis* of each subject, alternately from the left and right leg, using the Bergström-needle technique custom-adapted for manual suction^68^. Each biopsy on the same leg was taken approximately 1–2 cm distal from the previous one. The collected muscle samples were cleaned from blood and non-muscular tissues, snap frozen in liquid nitrogen and stored at -80 °C until further processing. The subjects were instructed to fast overnight before biopsy-days and consume a standardized meal consisting of a nutrients-drink (Fresubin Vanille, Fresenius; 20 g protein, 24.8 g carbs, 13.4 g fat, 1,260 kJ) 3 h before biopsies. All subjects were allowed to drink water *ad libitum*.

### Antibodies used for Western blotting

Primary: Desmin (D93F5), total (rabbit monoclonal IgG AB; #5332S; 1:1500; Cell Signaling Technology, Danvers, MA, USA); Desmin, total (rabbit polyclonal IgG AB; #PA5-16705; 1:3000; Thermo Fisher Scientific, Waltham, MA, USA); Desmin, phospho threonine 17 (rabbit polyclonal igG AB; #bs-5301R; 1:750; Bioss Antibodies, Woburn, MA, USA); Desmin, phospho serine 31 (rat monoclonal igG2b; #D375-3; 1: 2250; MBL International Corporation, Woburn, MA, USA); Desmin, phospho serine 60 (rabbit polyoclonal igG AB; #PA5-38837; 1:500; Thermo Fisher Scientific, Waltham, MA, USA); secondary: anti-rabbit IgG, HRP-linked (#7074S; 1:7500; Cell Signaling Technology, Danvers, MA, USA); anti-rat igG2b, HRP-linked (#PA1-84710; 1:7500; Thermo Fisher Scientific, Waltham, MA, USA).

### Antibodies used for immunohistochemistry

Primary: Desmin (D93F5), total (rabbit monoclonal IgG AB; #5332S; 1:1500; Cell Signaling Technology, Danvers, MA, USA); Desmin, phospho threonine 17 (rabbit polyoclonal igG AB; #bs-5301R; 1:100; Bioss Antibodies, Woburn, MA, USA); Desmin, phospho serine 31 (rat monoclonal igG2b AB; #D375-3; 1: 100; MBL International Corporation, Woburn, MA, USA); Desmin, phospho serine 60 (rabbit polyclonal igG AB; #PA5-38837; 1:50; Thermo Fisher Scientific, Waltham, MA, USA); MyHCI (mouse monoclonal IgG AB; #A4.951; 1:50; Developmental Studies Hybridoma Bank, Iowa City, IA, USA); MyHCI (mouse monoclonal IgG AB; #A4.840, 1:25; Developmental Studies Hybridoma Bank, Iowa City, IA, USA); Laminin (rabbit polyclonal IgG AB; #L9393; 1:50; Sigma-Aldrich, Darmstadt, Germany) Secondary: goat anti-mouse and goat anti-rabbit, polyclonal, biotinylated (#E0433 and #E0432; 1:400; Dako, Denmark); anti-rat igG2b, HRP-linked (#PA1-84710; 1:400; Thermo Fisher Scientific, Waltham, MA, USA); Alexa Flour 555 goat anti-mouse IgG (#A21238, 1:500, Invitrogen, Grand Island, NY, USA); Alexa 488 goat anti-mouse IgM (#A21426, 1:500, Invitrogen, Grand Island, NY, USA); Alexa 647 goat anti-rabbit IgG (#A21238, 1:400, Invitrogen, Grand Island, NY, USA).

### Tissue preparation

Muscle tissue was homogenized and lysed in a triton-X100-based buffer (#9803; Cell Signaling, Beverly, MA, USA) containing a protease and phosphatase inhibitor cocktail (#78429; 1:100; Thermo Scientific, Waltham, MA, USA) and Phenylmethanesulfonyl fluoride (PMSF; #P7626-1G; 1:100; Sigma Life Science, St. Louis, MO, USA) using a Precellys Lysing Kit (#P000918-LYSK0-A) and a Precellys Evolution (#P000062-PEVO0-A, both Bertin Technologies, Montigny le Bretonneux, France). Per ug tissue, 35 ul of buffer were added and homogenized for 3 cycles of 15 s at 5500 rpm with 2 min resting intervals on ice. After that, the samples incubated for 15 min on ice and afterwards were spun at 13,600 rpm for 10 min. The supernatant fraction of each sample was collected and the remaining pellet was again homogenized and lysed in a urea buffer (4 M Urea, Glycerol, 20% SDS, 1 M DTT, 1.5 M Tris). The volume of urea buffer added to the samples corresponds to 66.6% of the volume of the first buffer. Immediately after the buffer was added, the pellet was resuspended using the Precellys for 3 cycles of 20 s at 4500 rpm with 2 min resting intervals at room temperature. Thereafter, the samples incubated for 30 min at room temperature and then again were spun at 13,600 rpm for 10 min. Whole cell lysates were created with the same tissue by homogenizing and lysing in the urea buffer (same as above). Per ug tissue, 35 ul of urea buffer were added and processed like the pellet fraction.

Additionally, for the cleavage experiment, muscle tissue of 4 randomly selected participants were pooled and supernatant fractions were prepared according to the method described above with the addition of PMSF on the one hand, and without the addition of PMSF on the other, in order to enable a direct comparison.

### Western blotting

Protein concentrations of supernatant, pellet and whole cell lysates were determined via a Lowry test kit (BioRad). Before analysis, the lysates of each subject were diluted to a protein concentration of 1.5 mg/ml and suspended in a 4x Laemmli buffer (#1610747; BioRad Laboratories GmbH, Munich, Germany). Supernatant fractions were heated to 95 °C for 5 min, whole cell lysates for 1 min.

Equal amounts of protein (12 µg) for each subject and time point were separated on a 26-well, 4–12% BIS-TRIS Gel using a gel-casting system that worked with MOPS electrophoresis buffer. All used equipment and buffers were made by BioRad (BioRad Laboratories GmbH, Munich, Germany). After electrophoresis, the gel was transferred to a polyvinylidene difluoride (PVDF) membrane (GE Healthcare Life Science, Amersham, UK) by semidry blotting (Trans Blot Turbo, BioRad, Hercules, CA, USA) for 40 min (1.2 A, 25 Vmax). Equal sample loading and transfer were checked by staining the PVDF membrane with Ponceau S after transfer.

Membranes were blocked for one hour at room temperature in 5% nonfat dry milk dissolved in tris-buffered saline supplemented with 0.1% Tween20 (TBST), before being incubated with primary antibodies dissolved in 5% bovine serum albumin in TBST overnight at 4 °C. Then, membranes were washed three times with TBST and subsequently incubated with secondary antibodies diluted in TBST containing 5% nonfat dry milk for one hour at room temperature. After washing again, membranes were incubated for 3 min with an enhanced chemiluminescence assay (ECL-Kit, GE Healthcare Life Science, Amersham, UK) and automatically captured (ChemiDoc MP, BioRad, Hercules, CA, USA). Band densities were assessed semi-quantitatively using the Fiji software (v. 1.53t; National Institute of Health, New York, NY, USA).

### Immunohistochemistry

Frozen muscle samples were cut at -20 °C consecutive into 7 μm thick cross sections using a cryo-microtome (Leica CM 3050 S, Leica Microsystems, Nussloch, Germany) and thaw-mounted on polysine slides (VWR International, Langenfeld, Germany). All investigated time points of a subject were mounted on the same slide and all slides were stained in a single batch with the same antibody-dilution and incubation times to minimize variability in staining efficiency. For immunohistochemical staining of total and phosphorylated Desmin, slides were air-dried, fixed in -20 °C precooled Acetone for 8 min and air-dried again. Afterwards, the slides were blocked with 5% Bovine Serum Albumin (BSA) dissolved in Tris-buffered saline (TBS; 150 mM NaCl, 10 mM Tris-HCl, pH 7.6) for one hour before incubating the primary total and phosphorylated Desmin antibodies, each on a different slide, for 18 h at 4 °C. After that, the slides were washed with TBS and incubated with the matching biotinylated secondary antibody for one hour at room temperature. After washing once again, the slides (except _p_Des^S31^) were then incubated for one hour with a streptavidin biotinylated horseradish peroxidase complex (Amersham Biosciences, Uppsala, Sweden) diluted 1:400 in TBS and subsequently washed again. The staining was finalized by a 3,3′ diaminobenzidine (DAB) solution, which was incubated for 4 min. For determination of the fiber types, type I fibers were identified with A4.951 antibodies on the same slides and stained with a fluorochrome-linked secondary antibody (Alexa Flour 555 goat anti-mouse IgG). To confirm antibody specificity, control sections were incubated in TBS containing 0.8% BSA without primary antibodies. After dehydration on air over night, the stained sections were embedded in Entellan (Merck, Darmstadt, Germany) and covered with a coverslip (VWR International, Darmstadt, Germany).

For immunohistochemical staining used for fiber-type distribution and size, samples were stained for muscle fiber typing as well as cross sectional area (CSA) as described previously^69^. In short, samples were air dried for 30 min after taking them out of the freezer. After 5 min fixation in acetone, the cryo-sections were incubated for 30 min with anti-myosin heavy chain type 1 (A4.840) and anti-Laminin in a 0.05% Tween phosphate-buffered saline (PBS). Slides were then washed three times in the Tween/PBS solution. Appropriate secondary antibodies were then applied, GAMIgM Alexa 488 (A21426, Invitrogen, Grand Island, NY, 1:500) and GARIgG Alexa 647 (A21238, Invitrogen, 1:400) in combination with 4′,6-diamidino‐2‐phenylindole (DAPI; D1306, Invitrogen, 1:100) for 30 min. After a final triple washing with PBS, slides were mounted with Mowiol (Calbiochem).

### Quantification of sarcoplasmic desmin and phosphorylated desmin staining

Between 5 and 7 digital photos of each time point and subject were taken from stained cross-sections at 20-fold magnification via a light microscope (KS-300, Zeiss, Germany) coupled with a digital CCD camera (Sony, Tokyo, Japan). The target-specific staining intensity for each myofiber was quantified using the ImageJ software (v. 1.53t; National Institute of Health, USA) by selecting the sarcoplasmic region of the myofiber and its measurement through optical densitometry. For the fiber-type-specific analysis of total and phosphorylated Desmin, digital pictures of the same cross-sections were taken via a fluorescence microscope (ZOE Fluorescent Cell Imager, BioRad Laboratories GmbH, Munich, Germany). On average, 48 ± 14 type I and 46 ± 13 type II fibers were determined per time point and subject. In sum, 2030 type I and 1924 type II fibers were used for the analysis of the entire fiber population.

### Quantification of fiber-type distribution and size

Slides were viewed and automatically captured using 10x objective on a modified Olympus BX51 fluorescence microscope with a customized disk-spinning unit (DSU, Olympus, San Jose, CA, USA), computer-controlled excitation and emission filter wheels (Olympus), 3-axis high-accuracy computer-controlled stepping motor specimen stage (Grid Encoded Stage, Ludl Electronic Products, Hawthorne, NY, USA), ultra-high sensitivity monochrome electron multiplier CCD camera (C9100-02, Hamamatsu Photonics, Hamamatsu City, Japan) and controlling software (StereoInvestigator; MBF BioScience, Williston, VT, USA). Before analyses, slides were blinded for both, intervention and time point. All areas selected for analysis were free of ‘freeze fracture’ artefact, and care was taken such that longitudinal fibers were not used in the analysis. Quantitative analyses were performed using ImageJ software package (version 1.52p, National Institute of Health, MD, USA)^70^. On average, 83 ± 47 muscle fibers were analyzed per muscle biopsy sample collected to determine muscle fiber type distribution and CSA.

### Statistics

To check for the requirements of parametric tests, normal distribution was assessed for each time point and target with the Kolmogorov-Smirnov test. Where criteria were matched, the paired T-Test was used, otherwise the Wilcoxon matched-pairs signed rank test. Two-way ANOVA with Sidak’s corrections for multiple comparisons was used for analyzing changes in fiber type distribution and fiber cross sectional area. Data were normalized to baseline (pre 1, pre 14) through the division of the timepoints by the mean value of pre 1 or pre 14, respectively. To take into account that both male and female subjects were included and that there could be possible gender differences in the parameters examined, each statistic was also tested excluding the three female subjects (not shown). No relevant changes in the results were found. All statistics were carried out and graphs were created with the GraphPad Prism software (version 8.0.2). The significance level was set to *p* < 0.05.

## Electronic supplementary material

Below is the link to the electronic supplementary material.

Supplementary Material 1

## Data Availability

All data presented in the manuscript can be made available upon request from the corresponding author on reasonable interest.
