# Supplementary material for: Acute resistance exercise and training reduce desmin phosphorylation at serine 31 in human skeletal muscle, making the protein less prone to cleavage
Source: Sci Rep. 2024 Nov 14;14:28079. doi: 10.1038/s41598-024-79385-0 (PMC11564833; doi:10.1038/s41598-024-79385-0)
Supplement: Supplementary file 1 — Supplementary Material 1 [file 41598_2024_79385_MOESM1_ESM.pdf]

Supplemental material:

Original images on which those shown in the manuscript are based.

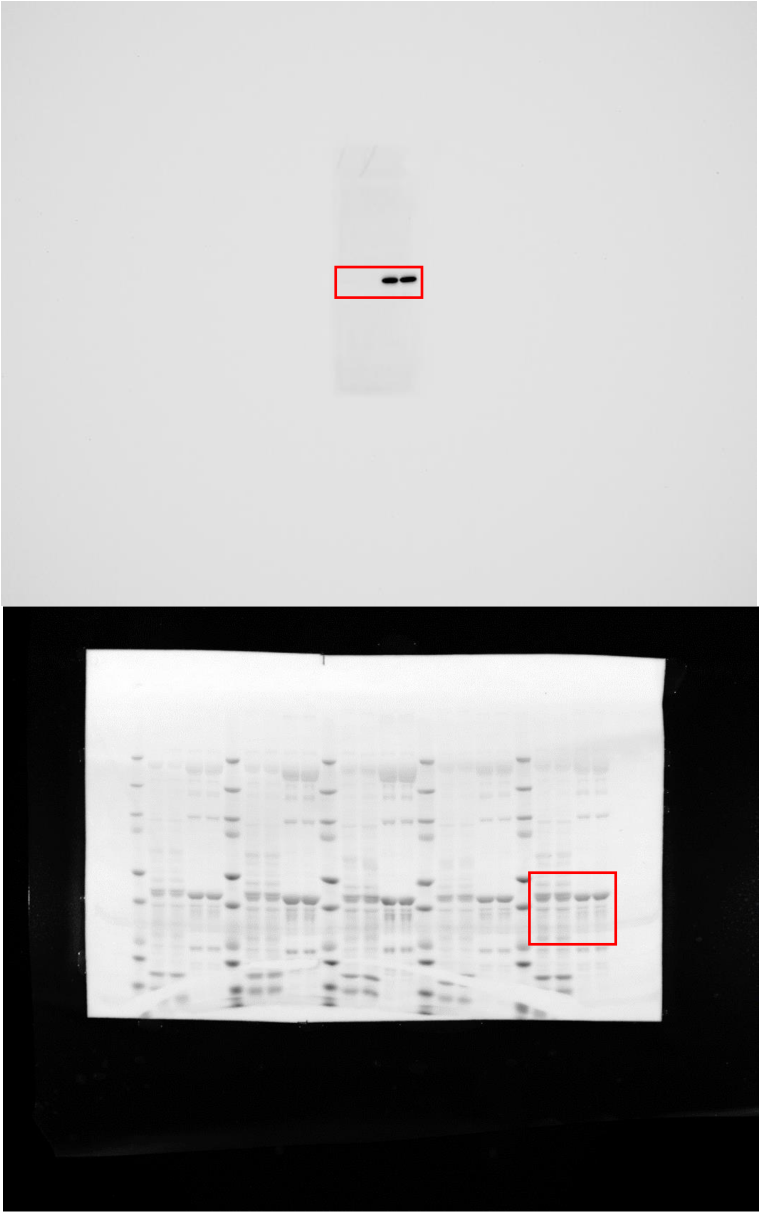

Figure S1 Original images of antibody incubated (top) and Ponceau S-stained western blot PVDF membrane (below) corresponding to manuscript image 1A.

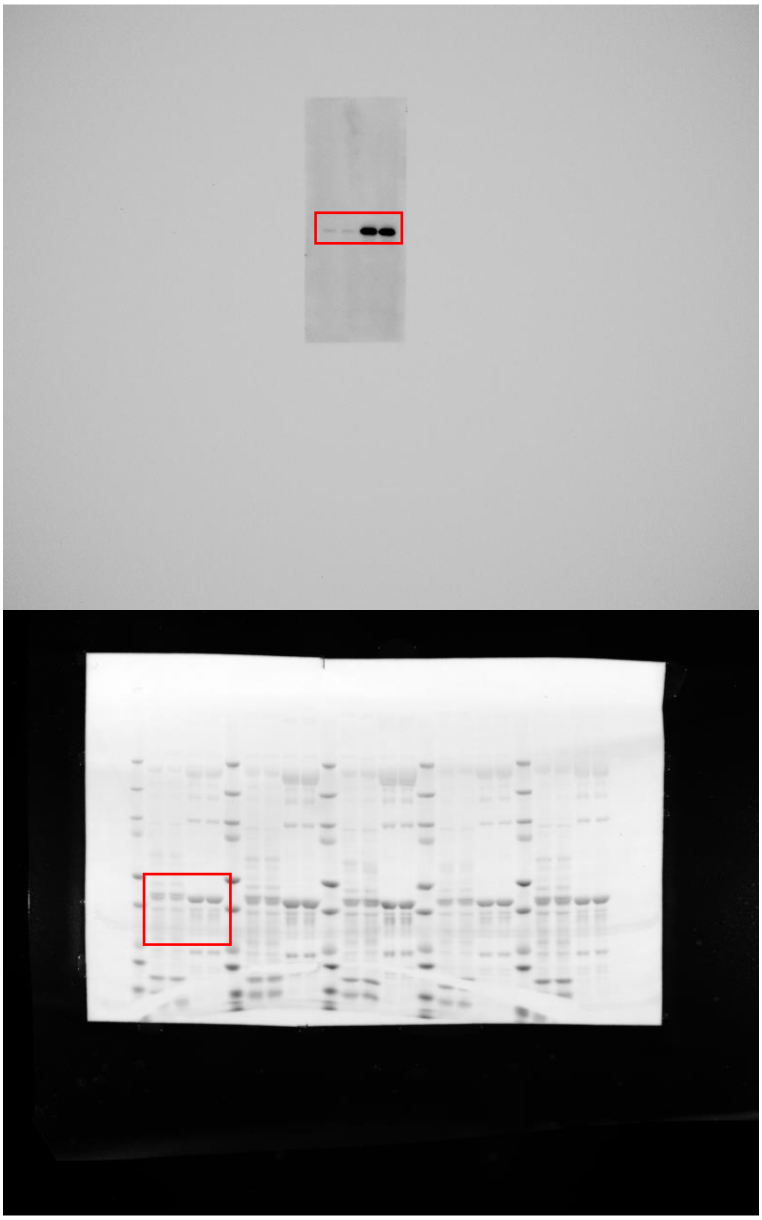

Figure S2 Original images of antibody incubated (top) and Ponceau S-stained western blot PVDF membrane (below) corresponding to manuscript image 1B.

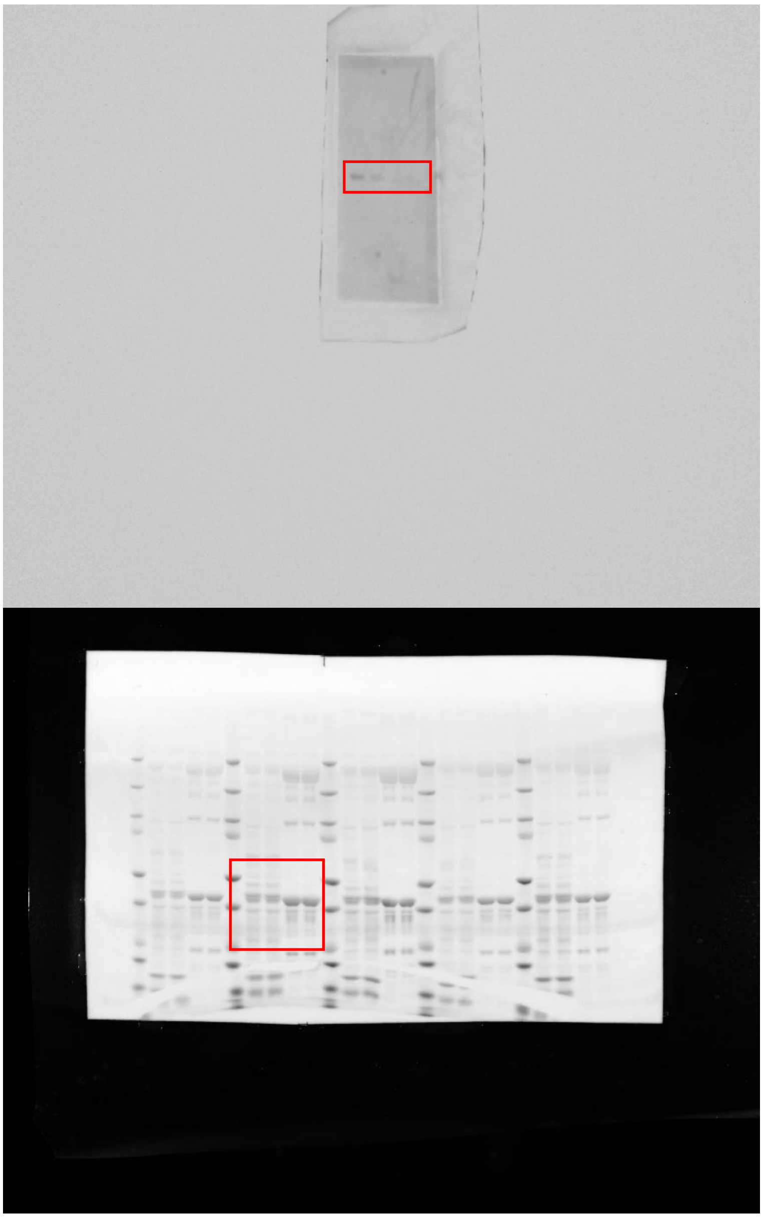

Figure S3 Original images of antibody incubated (top) and Ponceau S-stained western blot PVDF membrane (below) corresponding to manuscript image 1C.

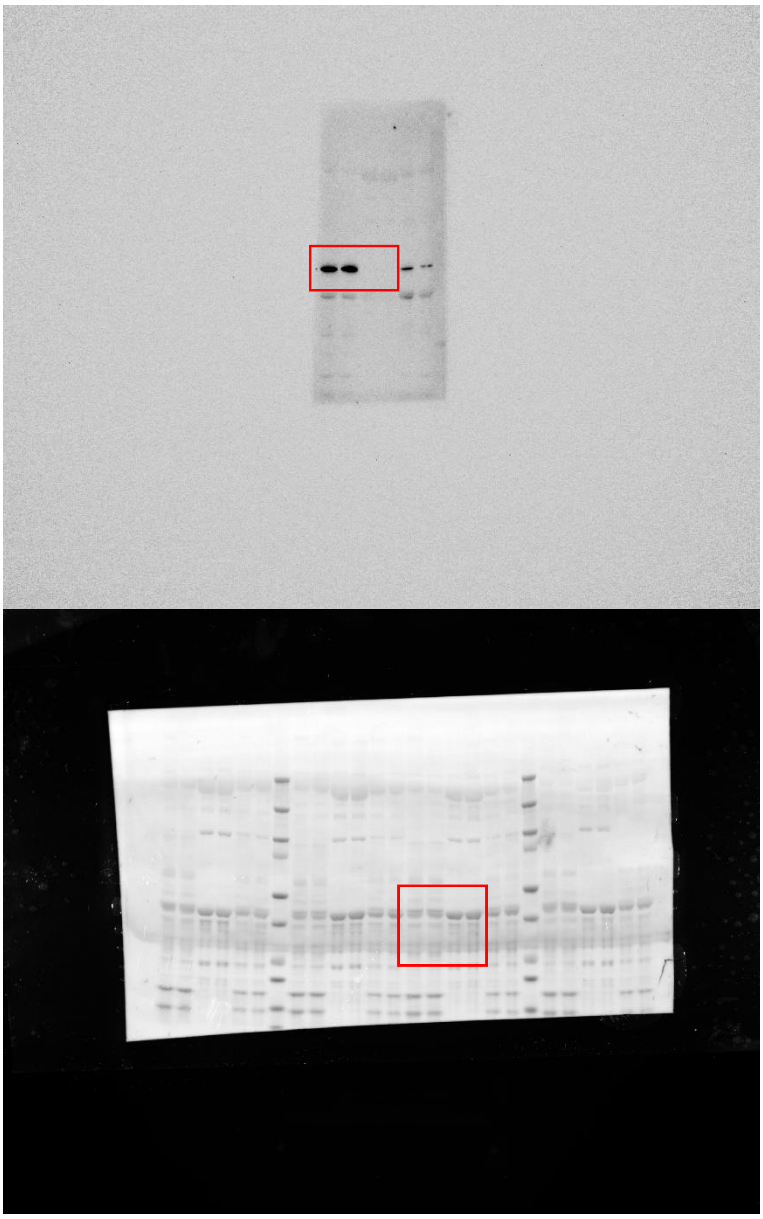

Figure S4 Original images of antibody incubated (top) and Ponceau S-stained western blot PVDF membrane (below) corresponding to manuscript image 1D.

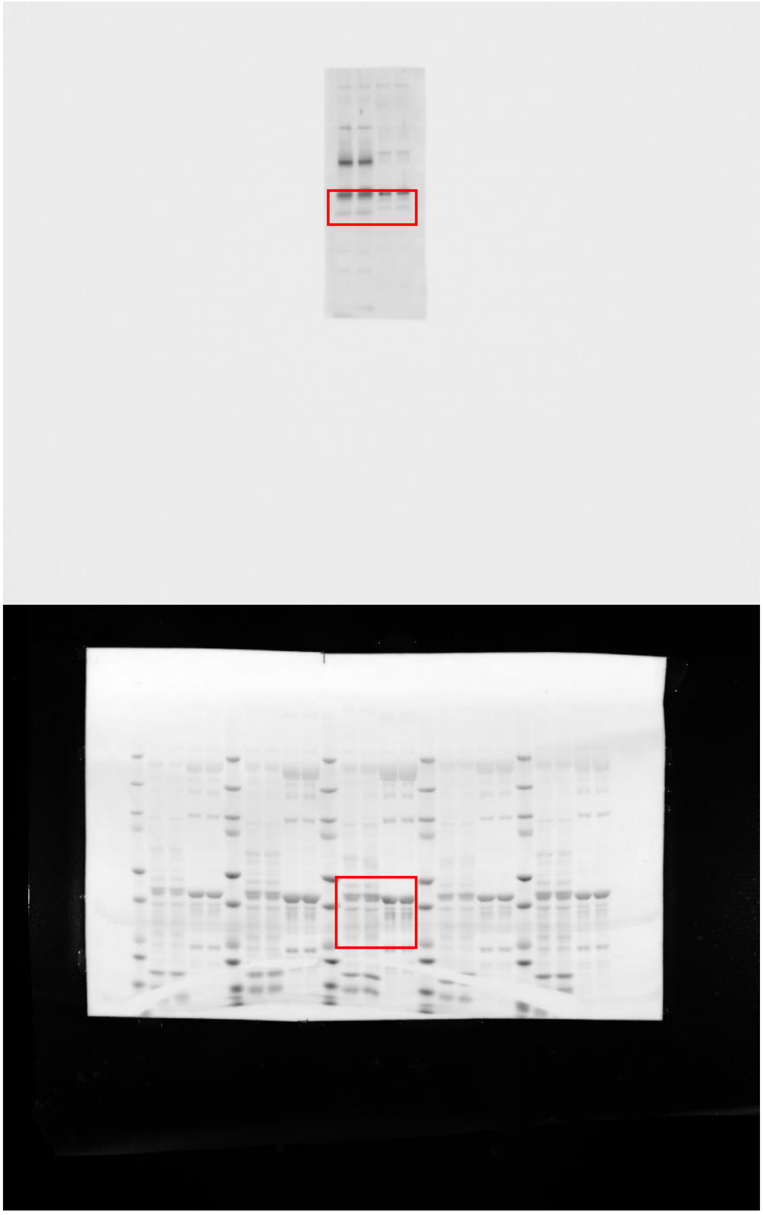

Figure S5 Original images of antibody incubated (top) and Ponceau S-stained western blot PVDF membrane (below) corresponding to manuscript image 1E.

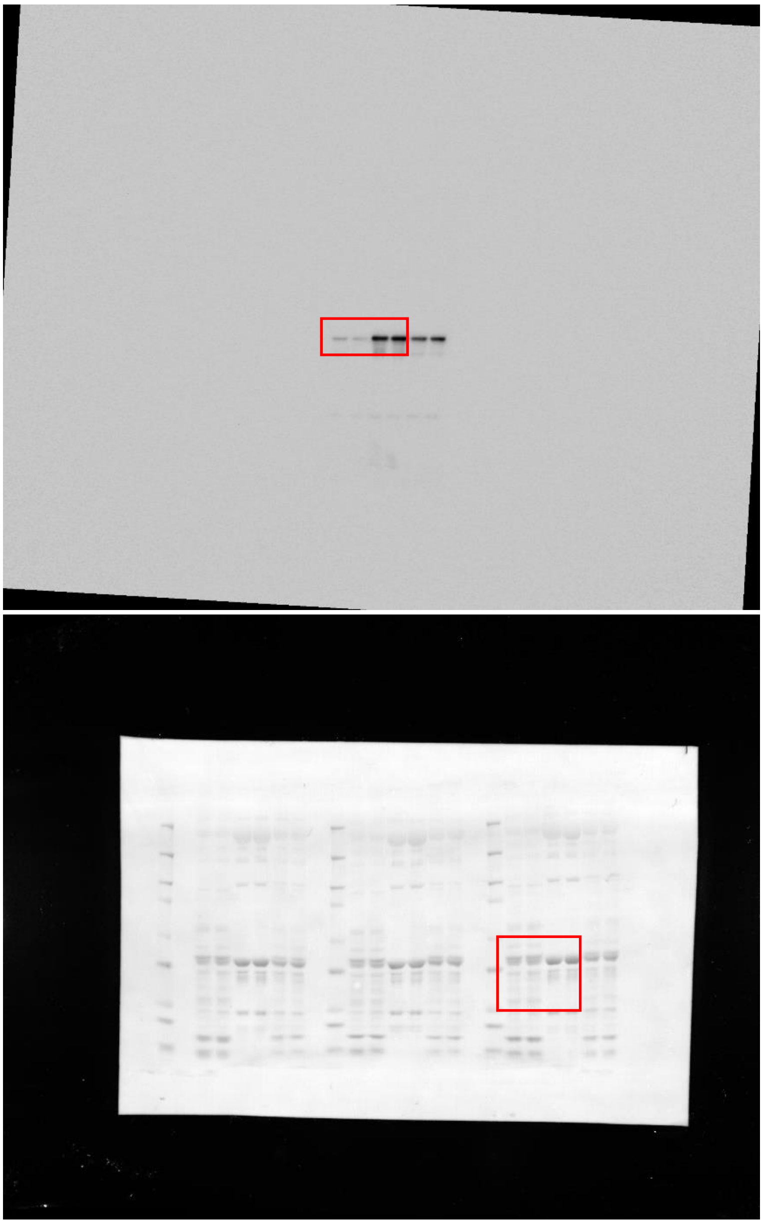

Figure S6 Original images of antibody incubated (top) and Ponceau S-stained western blot PVDF membrane (below) corresponding to manuscript image 1F.

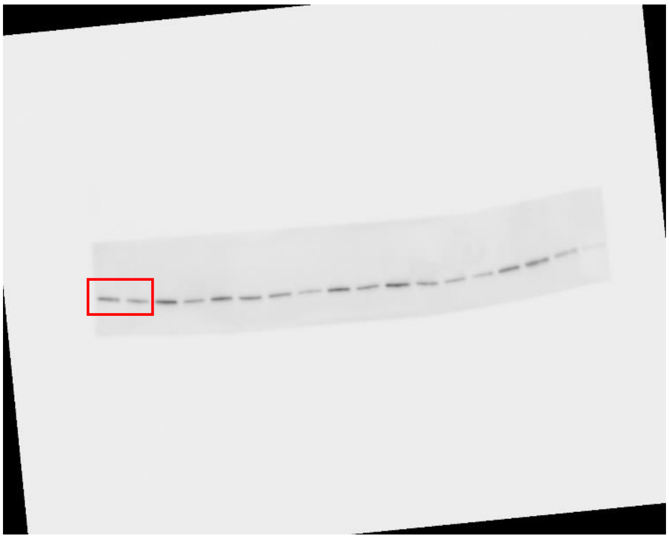

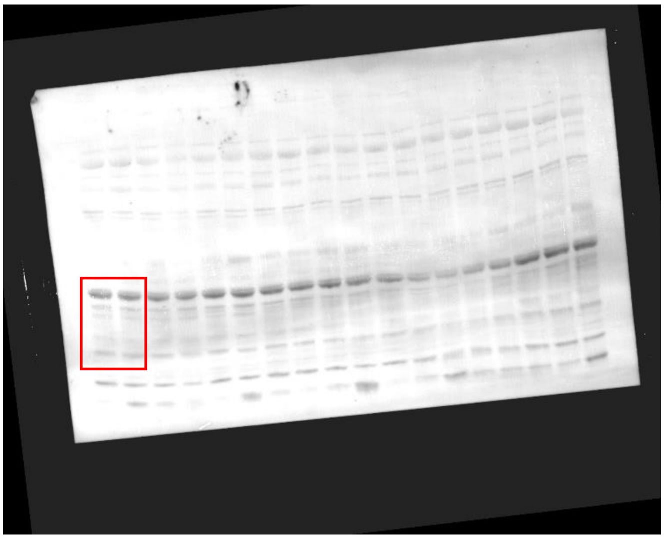

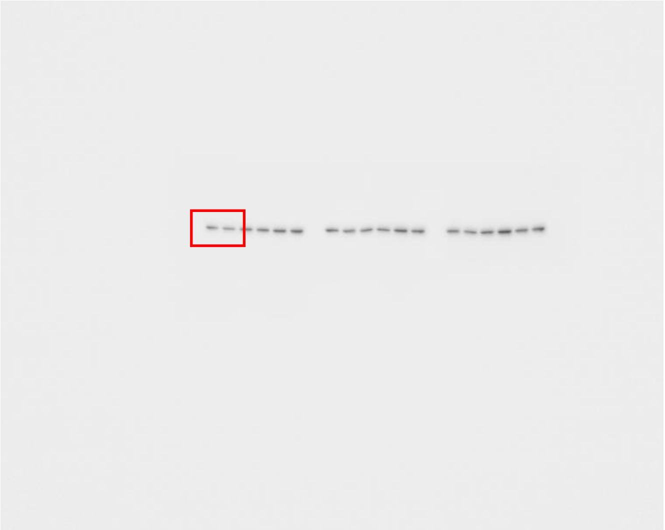

Figure S7 Original images of antibody incubated (top: pDes S31; below: total) and Ponceau S-stained western blot PVDF membrane (middle) corresponding to manuscript image 2B. Ponceau S of total is not illustrated.

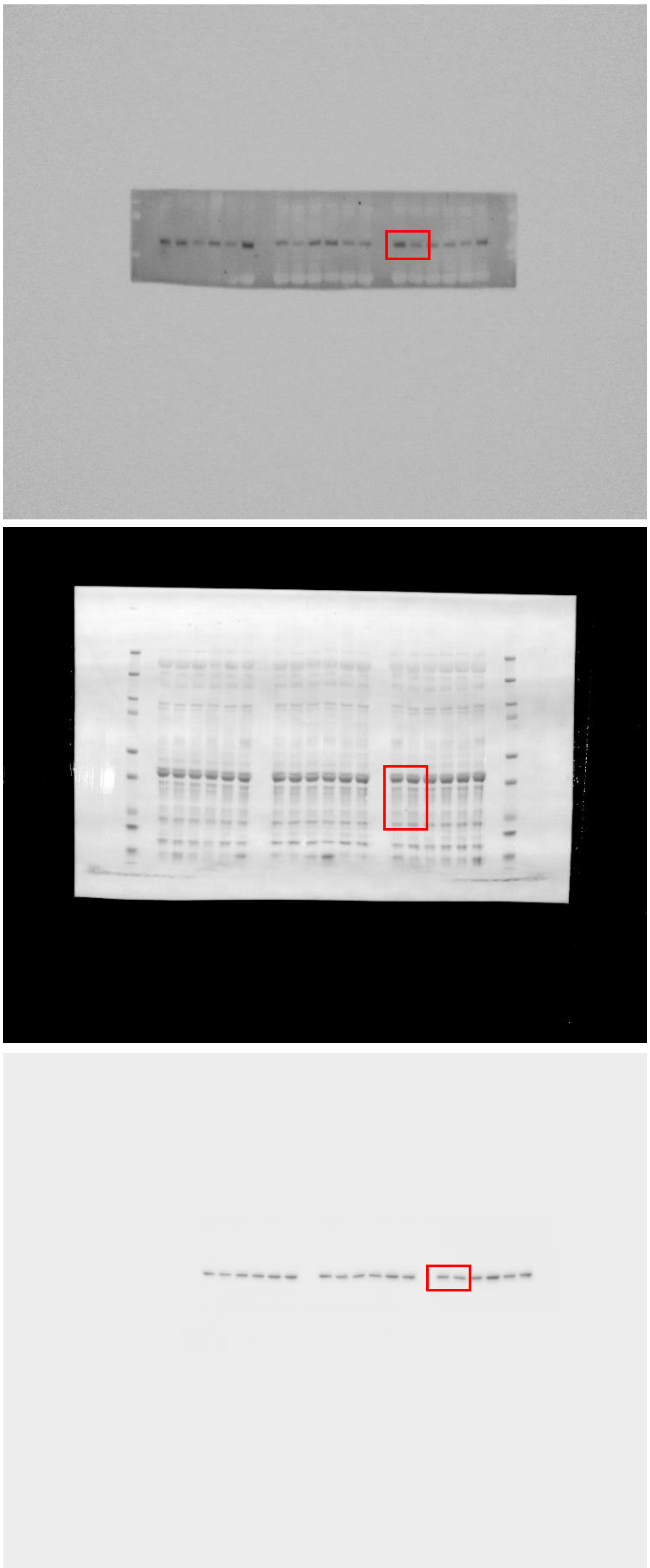

Figure S8 Original images of antibody incubated (top: pDes S60; below: total) and Ponceau S-stained western blot PVDF membrane (middle) corresponding to manuscript image 2C. Ponceau S of total is not illustrated.

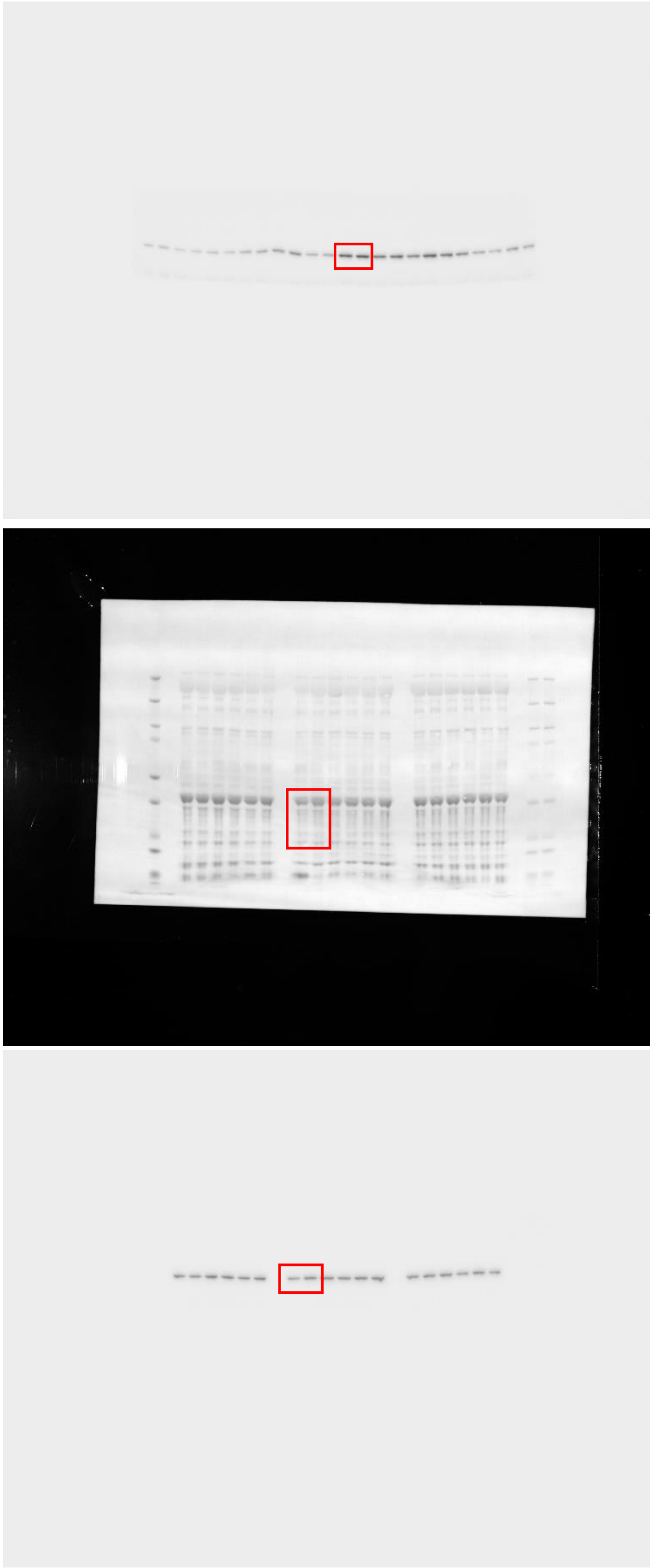

Figure S9 Original images of antibody incubated (top: pDes T17; below: total) and Ponceau S-stained western blot PVDF membrane (middle) corresponding to manuscript image 2D. Ponceau S of pT17 is not illustrated.

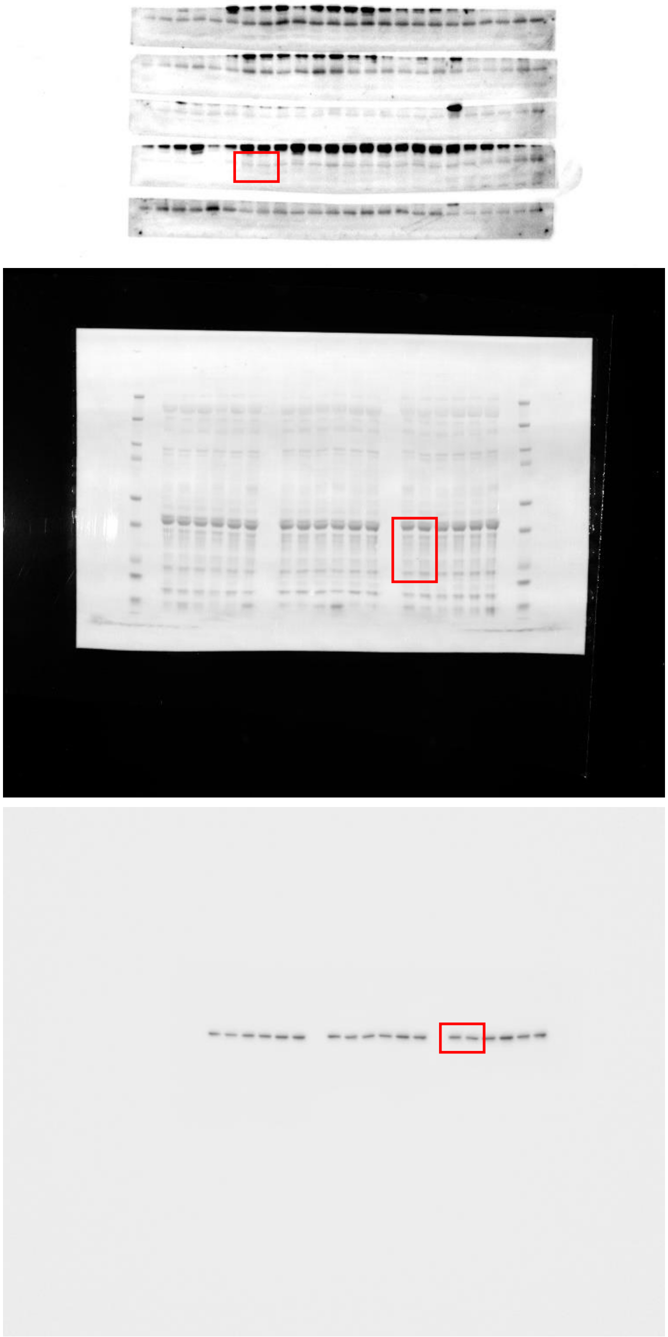

Figure S10 Original images of antibody incubated (top: pDes T76_77; below: total) and Ponceau S-stained western blot PVDF membrane (middle) corresponding to manuscript image 2E. Ponceau S of pT76_77 is not illustrated.

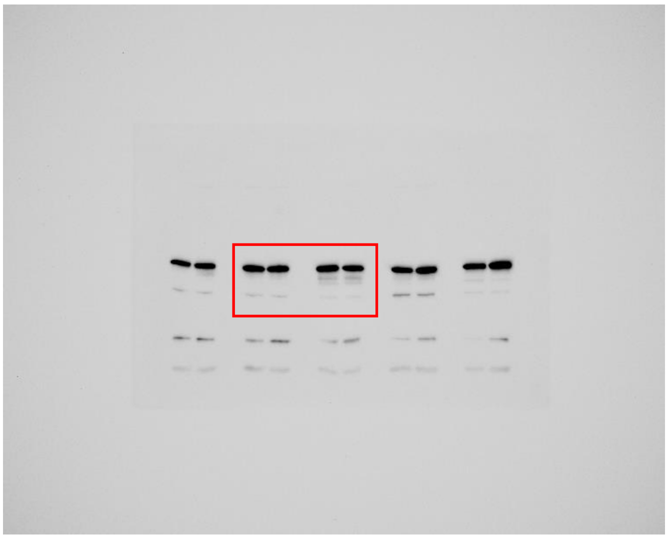

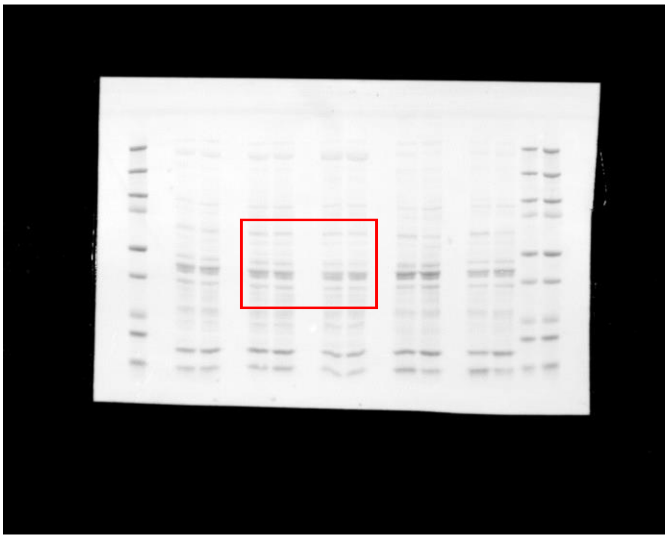

Figure S11 Original images of antibody incubated (top) and Ponceau S-stained western blot PVDF membrane (middle) corresponding to manuscript image 3A.

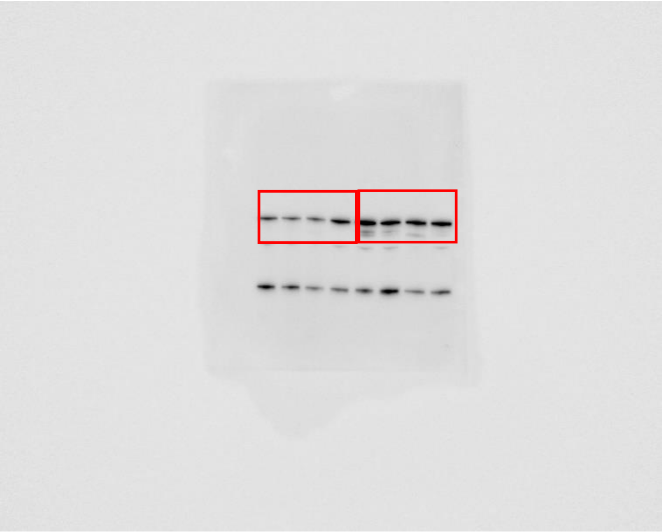

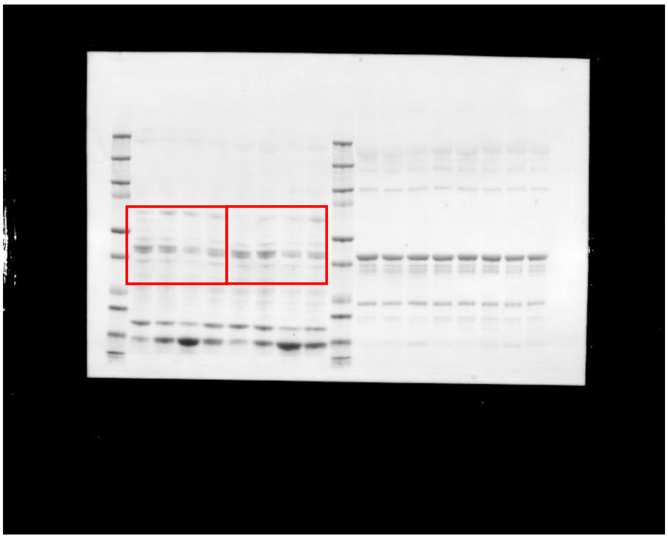

Figure S12 Original images of antibody incubated (top) and Ponceau S-stained western blot PVDF membrane (middle) corresponding to manuscript image 3B.

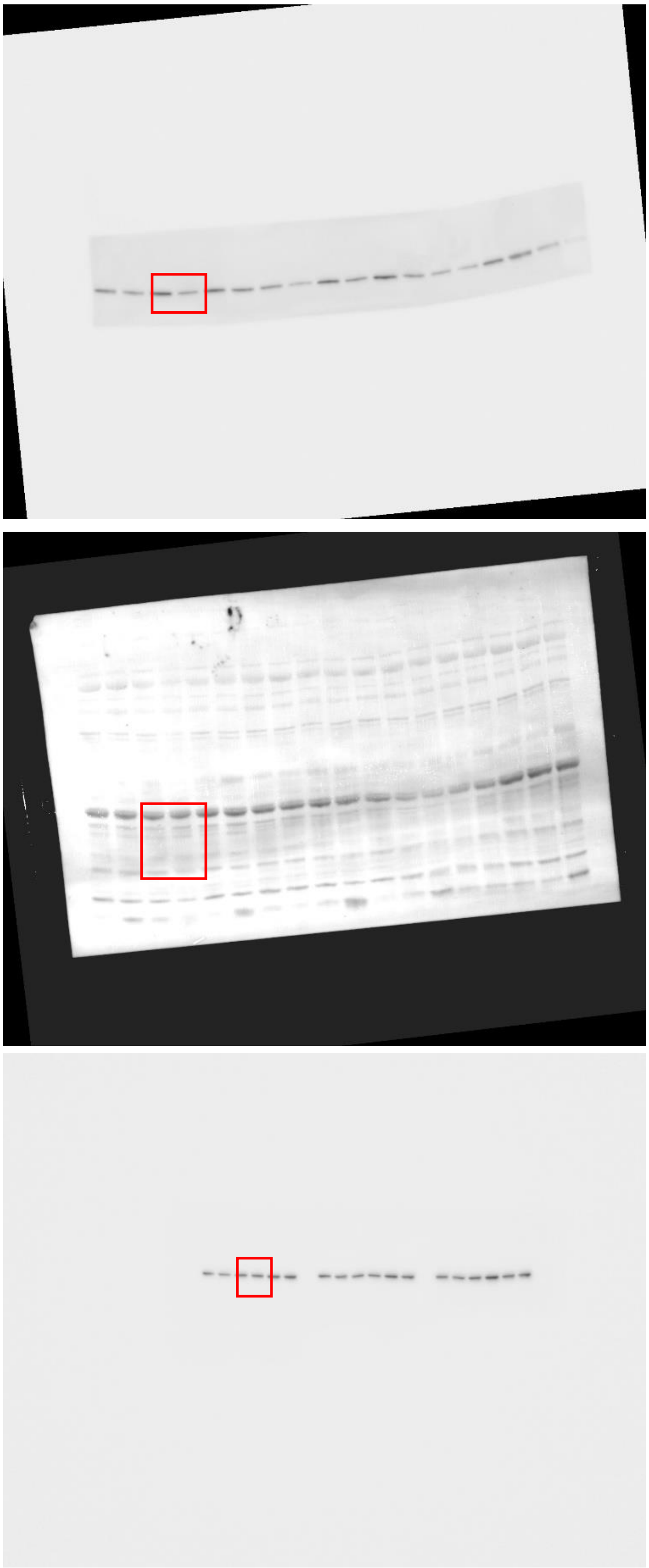

Figure S13 Original images of antibody incubated (top: pDes S31; below: total) and Ponceau S-stained western blot PVDF membrane (middle) corresponding to manuscript image 5B. Ponceau S of total is not illustrated.

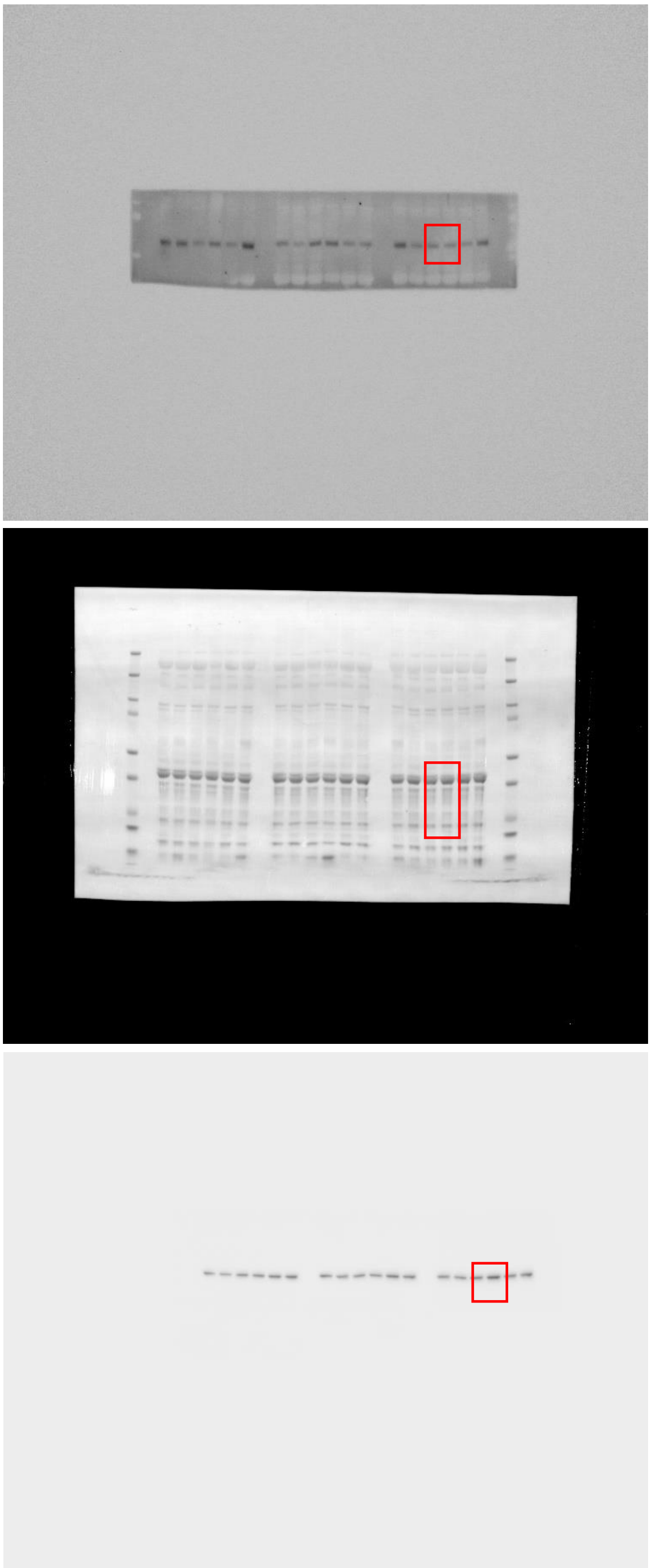

Figure S14 Original images of antibody incubated (top: pDes S60; below: total) and Ponceau S-stained western blot PVDF membrane (middle) corresponding to manuscript image 5C.

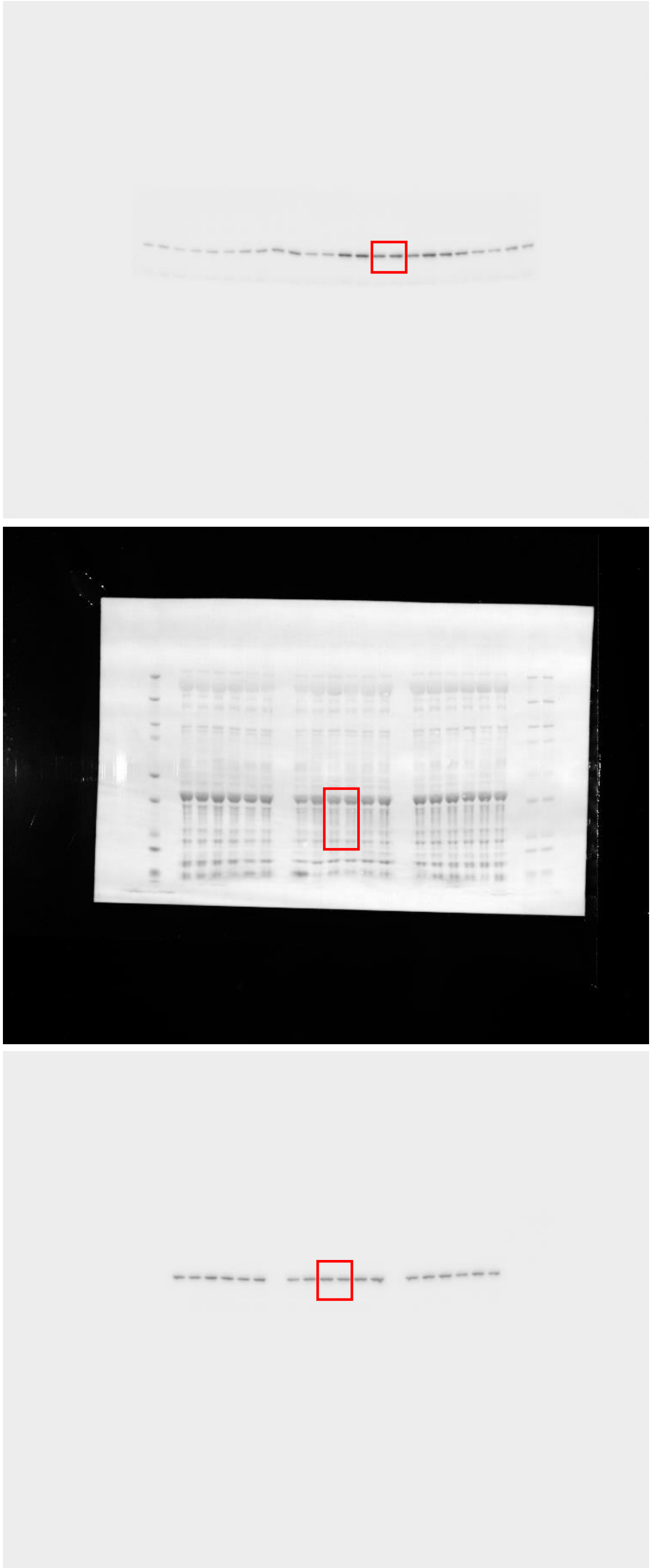

Figure S15 Original images of antibody incubated (top: pDes T17; below: total) and Ponceau S-stained western blot PVDF membrane (middle) corresponding to manuscript image 5D. Ponceau S of pT17 is not illustrated.

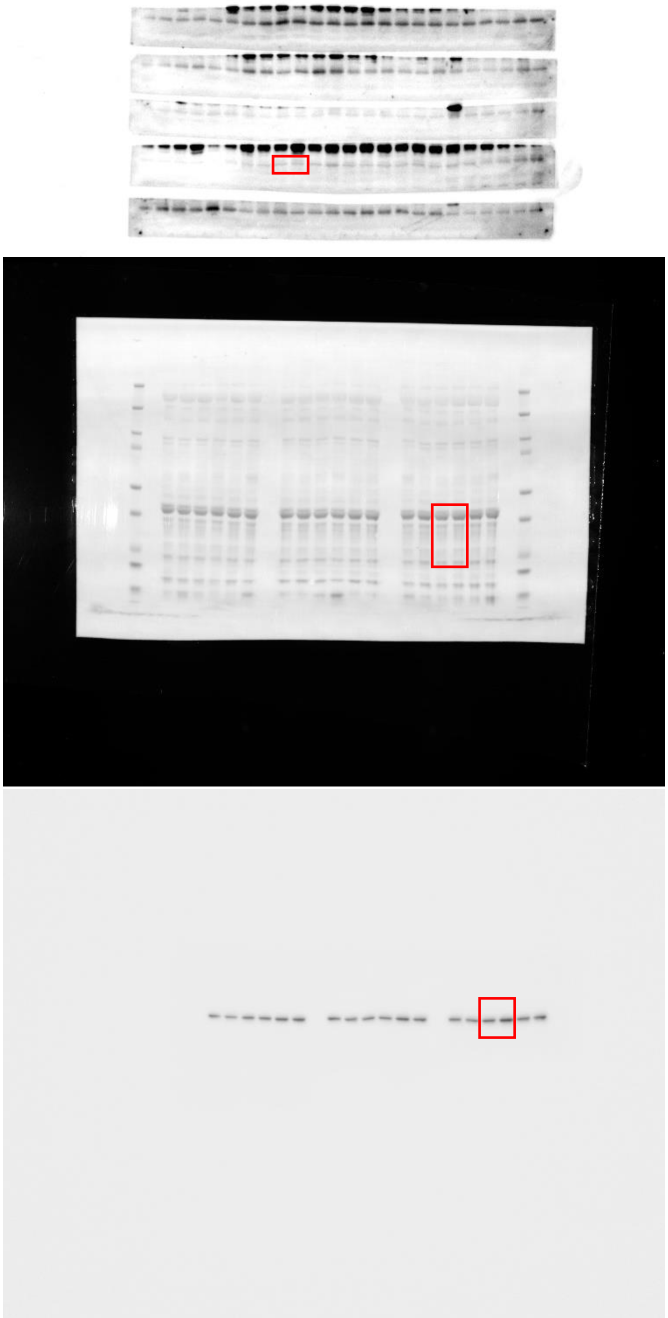

Figure S16 Original images of antibody incubated (top: pDes T76_77; below: total) and Ponceau S-stained western blot PVDF membrane (middle) corresponding to manuscript image 5E. Ponceau S of pT76_77 is not illustrated.

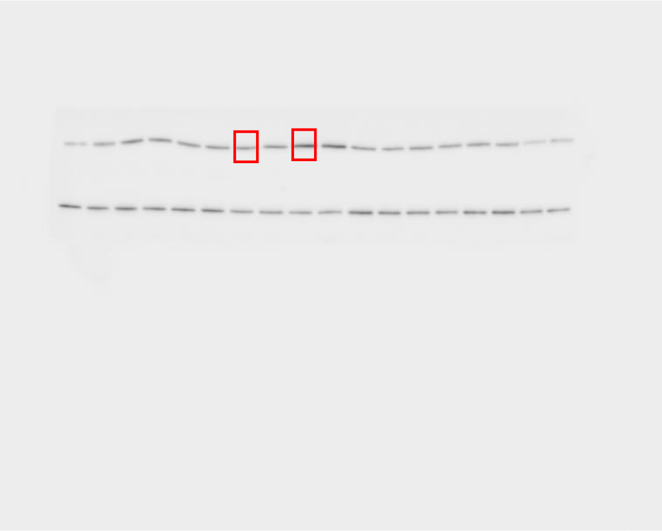

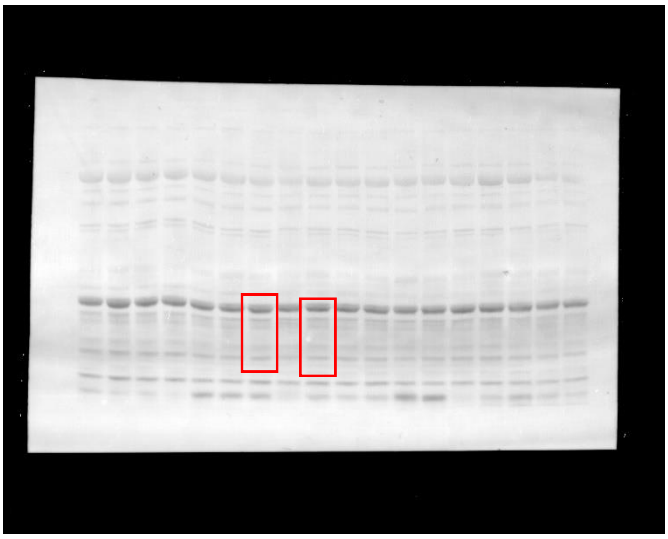

Figure S17 Original images of antibody incubated (top: total Des) and Ponceau S-stained western blot PVDF membrane (below) corresponding to manuscript image 6B.

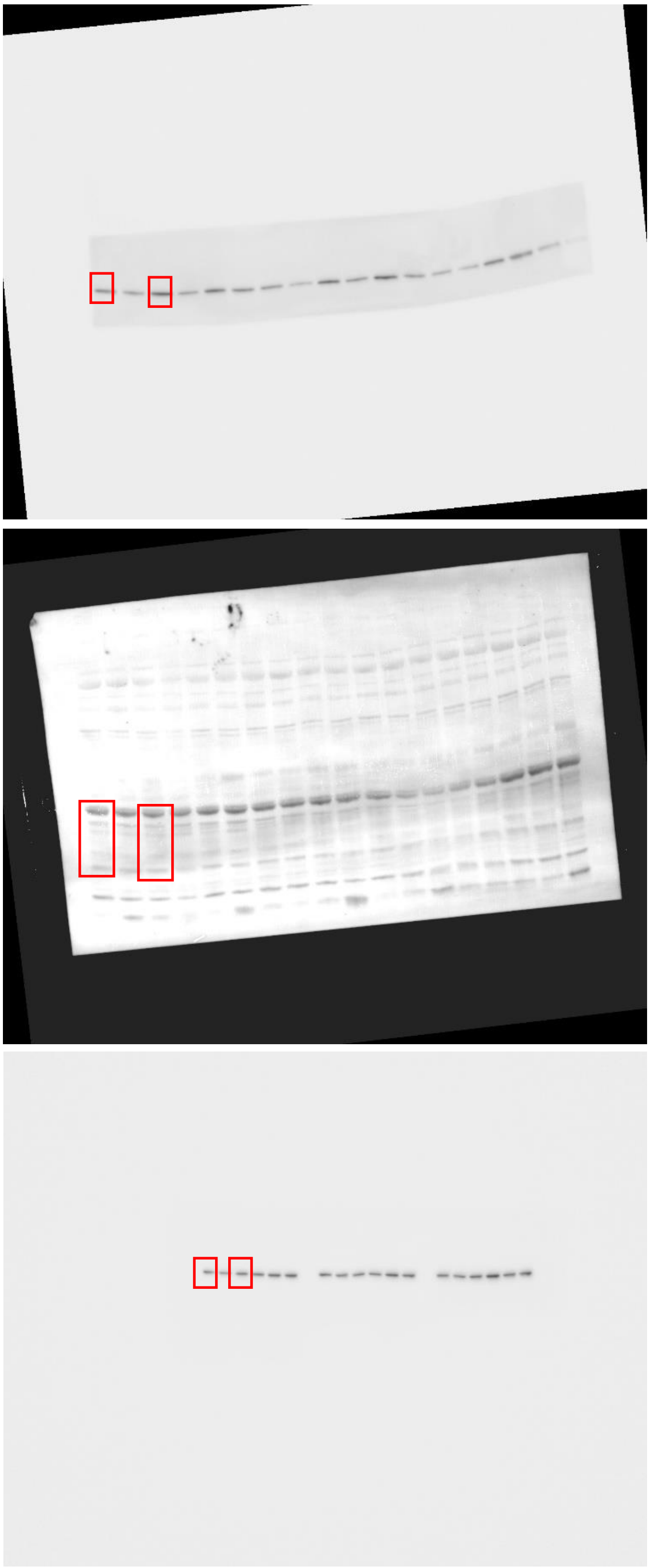

Figure S18 Original images of antibody incubated (top: pDes S31; below: total) and Ponceau S-stained western blot PVDF membrane (middle) corresponding to manuscript image 6C. Ponceau S of total is not illustrated.

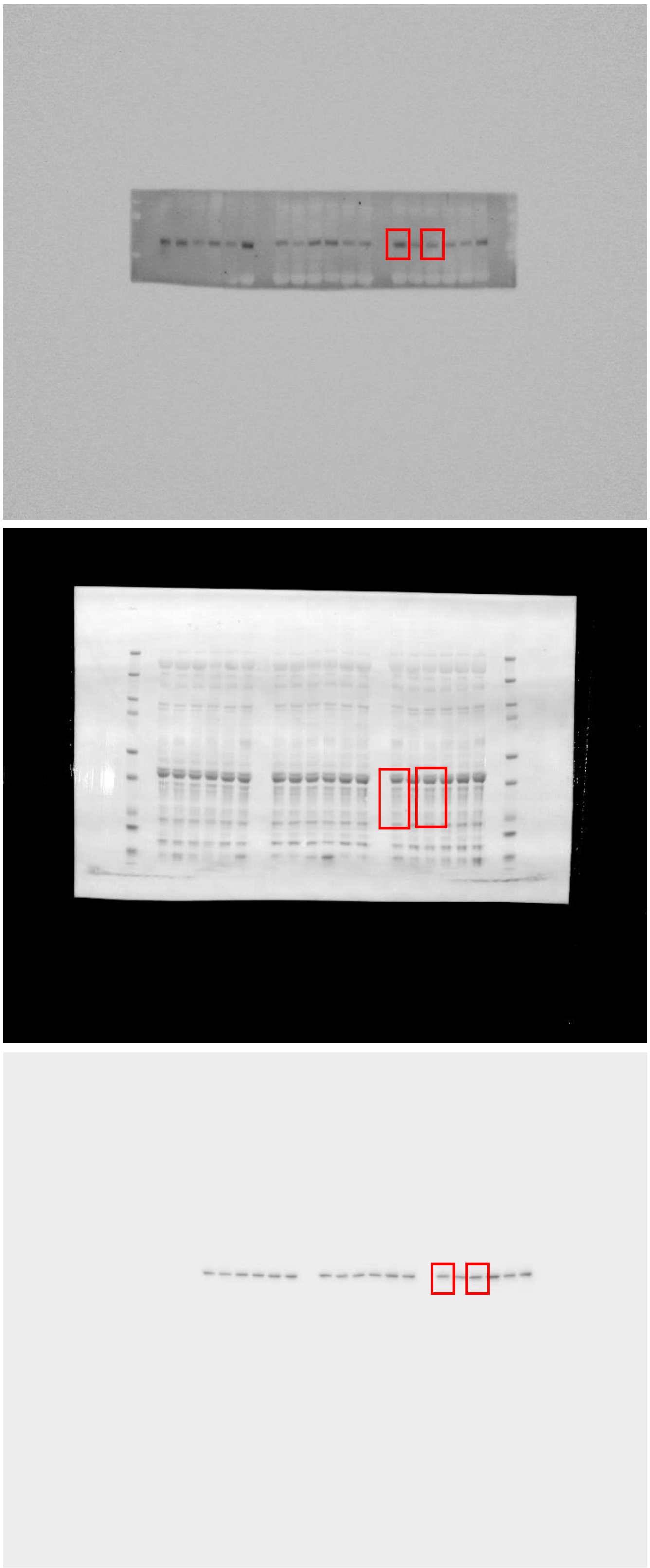

Figure S19 Original images of antibody incubated (top: pDes S60; below: total) and Ponceau S-stained western blot PVDF membrane (middle) corresponding to manuscript image 6D.

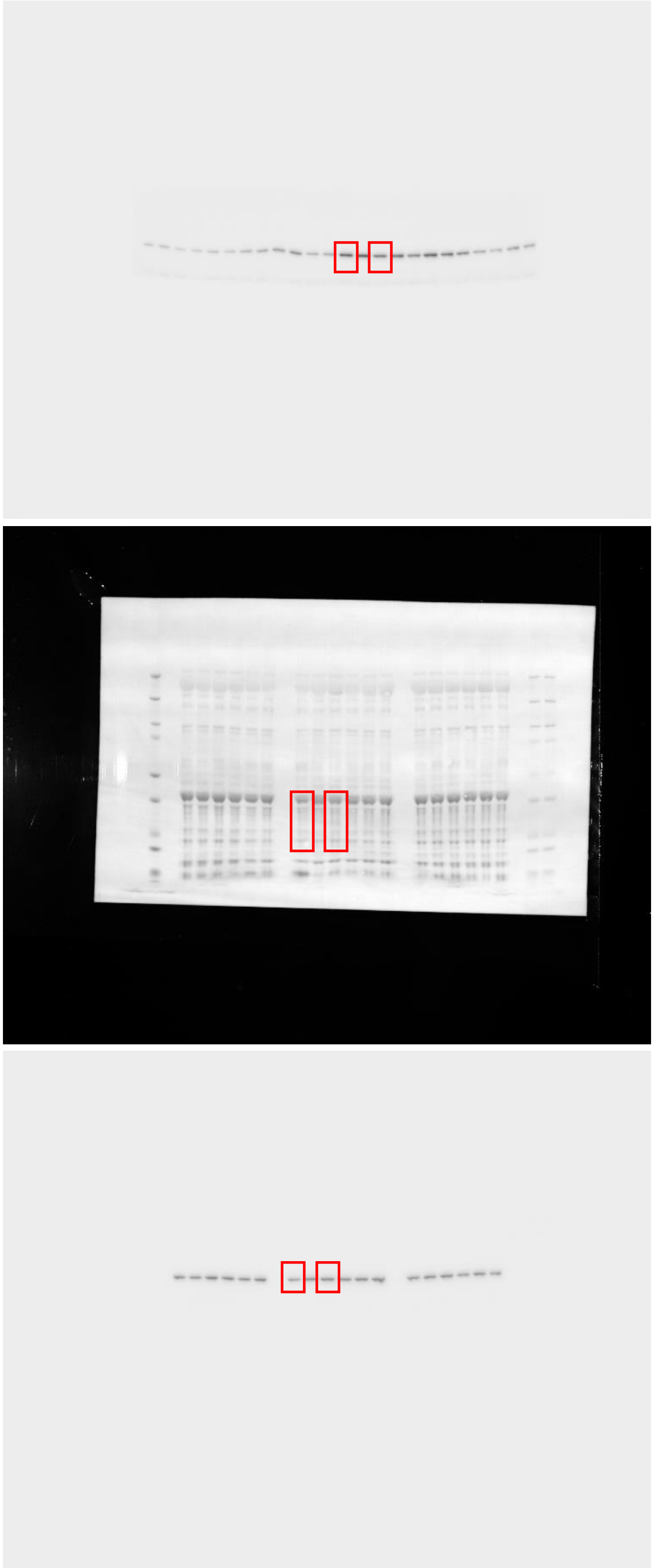

Figure S20 Original images of antibody incubated (top: pDes T17; below: total) and Ponceau S-stained western blot PVDF membrane (middle) corresponding to manuscript image 6F. Ponceau S of pT17 is not illustrated.

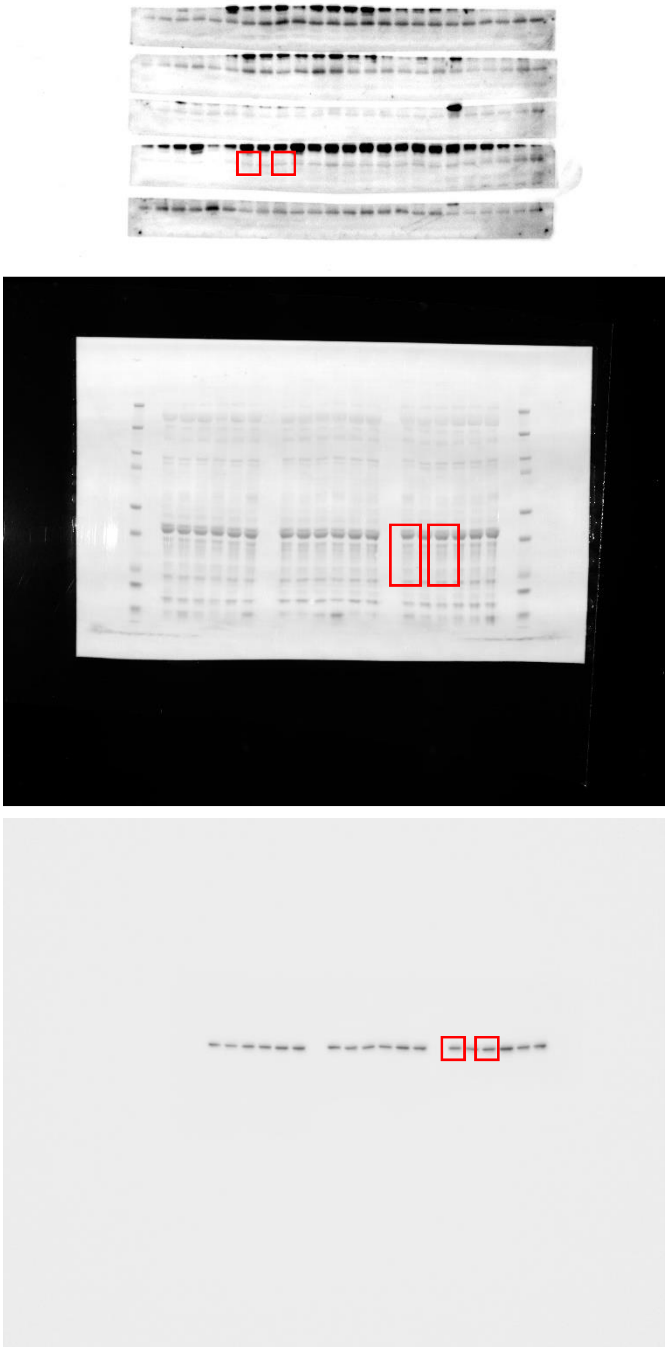

Figure S21 Original images of antibody incubated (top: pDes T76_77; below: total) and Ponceau S-stained western blot PVDF membrane (middle) corresponding to manuscript image 6F. Ponceau S of pT76_77 is not illustrated.
